# The effects of daytime napping on psychophysiological measures in physically active individuals and athletes: A systematic review, meta-analysis, and meta-regression, with assessment of the certainty of evidence

**DOI:** 10.5114/biolsport.2026.153310

**Published:** 2025-08-13

**Authors:** Omar Boukhris, Khaled Trabelsi, Haresh Suppiah, Haitham Jahrami, Matthew Driller

**Affiliations:** 1 Sport, Performance, and Nutrition Research Group, School of Allied Health, Human Services, and Sport, La Trobe University, Melbourne, Australia; 2 SIESTA Research Group, School of Allied Health, Human Services and Sport, La Trobe University, Melbourne, Victoria, Australia; 3 Research laboratory, Education, Motricity, Sport and Health (EM2S), LR15JS01, High Institute of Sport and Physical Education, University of Sfax, Tunisia; 4 Department of Movement Sciences and Sports Training, School of Sport Science, The University of Jordan, Amman-Jordan; 5 Department of Psychiatry, Ministry of Health, Manama, Bahrain; 6 Department of Psychiatry, College of Medicine and Medical Sciences, Arabian Gulf University, Manama, Bahrain

**Keywords:** Nap, Sleep, Sport, Recovery, Fatigue

## Abstract

To synthesise the impacts of napping following normal sleep (NS) or deprived sleep on psychophysiological measures in physically active individuals and athletes. This systematic review and metaanalysis utilized nine databases, including Web of Science, PubMed/MEDLINE, Scopus, SPORTDiscus, Embase, ProQuest Central, Cochrane Library, PsycInfo, and SciElo, to evaluate the effects of napping in physically active individuals and athletes, focusing on psychophysiological measures. The risk of bias in the included studies was assessed using the Cochrane Collaboration’s RoB 2.0 tool, while the certainty of evidence (CoE) was evaluated using the GRADE (Grading of Recommendations, Assessment, Development, and Evaluations) approach. In the 35 studies, 489 participants (athletes or physically active) were studied. Following NS, napping significantly reduced total mood score (standardized mean difference (SMD)=0.61), fatigue (SMD=0.91), rating of perceived exertion (RPE) both during (SMD=1.62) and immediately after exercise (SMD=1.11). Additionally, napping significantly improved perceived recovery (SMD=1.66). There were no significant effects of napping on sleepiness (SMD=1.09), muscle soreness (SMD=1.57), heart rate during exercise (SMD=0.62), and temperature (SMD=0.66). Similarly, following sleep deprivation, there were no significant effects of napping on sleepiness (SMD=1.03) and fatigue (SMD=0.79). The CoE was rated as moderate for RPE during and after exercise following NS, while it was low to very low for the remaining outcomes. Napping has been found to positively impact only fatigue, mood states, perceived exertion, and recovery following NS in physically active individuals and athletes. The low-to-very low CoE requires cautious interpretation, highlighting the need for further napping studies implementing robust methodologies.

## INTRODUCTION

Without adequate sleep, athletes may struggle to reach their full potential [1]. In fact, sleep is essential for both physical and mental performance, playing a critical role in recovery, cognitive function, and overall well-being [2–4]. Inadequate sleep can impair reaction time, decision-making, mood, and physical recovery, thereby compromising athletic performance [2]. This is particularly relevant considering that athletes are often exposed to factors that negatively impact sleep, such as high training loads, early morning training sessions, jet lag, travel, and night-time competition [2, 5]. In light of these challenges, short daytime sleep (i.e., napping) has emerged as a potential strategy to optimize athletic performance. Napping has been shown to be beneficial not only after a disrupted or disturbed night’s sleep but also following normal sleep (NS) (normal sleep means getting 7–9 hours of sleep per night) [6–8]. Evidence suggests that naps should ideally range between 25 and 90 minutes for practicality and effectiveness in sport setting [8, 9], with longer naps potentially providing greater benefits due to their ability to include more sleep time or more deep sleep, facilitating both physical and cognitive recovery [10, 11]. For optimal results, naps should be taken between 13:00 and 16:00. In this context, two recent systematic reviews with meta-analyses [9, 12] and three systematic reviews [8, 13, 14] have reported that daytime napping can enhance physical performance, both in cases of sleep deprivation (SD) and after NS [8, 12–14], supporting its role in athletic performance optimisation.

While physical performance improvements have been well-documented [8, 9, 12], understanding how napping influences psychophysiological responses, key determinants of athletic success, remains critical. Psychophysiological measures, such as the perception of fatigue, mood state, well-being, and the athlete’s psychological and physiological state, are crucial to understanding the mechanisms behind performance improvement [3, 4, 15, 16]. The results of studies examining the effects of napping on psychophysiological measures are contradictory. For example, some research has shown that daytime napping improved perceptual and physiological measures following NS [11, 17] or SD [18, 19]. In contrast, other studies have reported no changes in perceptual and physiological measures after NS [20–22] or SD [23]. However, despite the increasing interest in the benefits of napping, a comprehensive review focusing on the effects of napping on these specific psychophysiological measures in athletes is lacking. A systematic review and meta-analysis can provide a robust assessment of these effects, revealing the magnitude and consistency of the impact, identifying potential moderators, and highlighting gaps in current research [24, 25].

Therefore, the objective of this research was to systematically review the evidence from experimental studies (e.g., randomised, nonrandomised trials, interventional trials) and, where possible, conduct meta-analyses to examine the impacts of afternoon napping following NS or SD, on psychophysiological measures in athletes and physically active individuals.

## MATERIALS AND METHODS

The Preferred Reporting Items for Systematic Reviews and Meta-Analysis (PRISMA 2020) guidelines were followed for conducting the present systematic review and meta-analysis [26–28]. The PRISMA 2020 checklist is provided in the Supplementary File. The protocol of this review was registered in the Open Science Framework database (https://doi.org/10.17605/OSF.IO/82KZF). PRISMA checklist is presented in Table S1.

### Information sources and search

The Web of Science, PubMed/MEDLINE, Scopus, SPORTDiscus, Embase, ProQuest Central, Cochrane Library, PsycInfo, and SciElo databases were searched without applying any language restriction, filters or time limits from inception to 23 June 2024. The keywords used for the search strategy were associated with diurnal sleep and psychophysiological measures (Table 1). Medical subject headings (MeSH) were utilized in appropriate cases. Additionally, we reviewed the reference lists of the included studies and investigated related citations from other journals, in addition to searching personal files. Additionally, one author was contacted to obtain missing raw data essential for our analysis, but he did not respond to our inquiries; therefore, we could not include this study in our analysis.

**TABLE 1:** The PICO(S) strategy followed in the current systematic review and meta-analysis.

Search strategy item	Details
**Keywords**	(napping OR nap OR “daytime nap” OR siesta OR “daytime sleep”) AND (“heart rate” OR “heart rate variability” OR “blood pressure” OR “body temperature” OR “biochemical reponses” OR “hematological responses” OR “muscle damage” OR inflammation OR lactate OR “blood lactate” OR “biomarkers of antioxidant defense” OR “oxidative stress” OR “Electroencephalography” OR Electroencephalogram OR “cardiac output” OR “maximal oxygen uptake” OR ${\dot{\text{V}}\text{O}}_{2\max}$ OR immunity OR physiology OR “physiological responses” OR “brain activity” OR “perceived effort” OR “perceived exertion” OR “perceived fatigue” OR fatigue OR “rating of perceived exertion” OR RPE OR “perceived recovery” OR “perceived recovery status” OR “muscle soreness” OR “delayed onset muscle soreness” OR sleepiness OR “daytime sleepiness” OR “mood state” OR mood OR feeling OR emotion OR depression OR stress OR confusion OR anxiety OR tension OR anger OR motivation OR satisfaction OR “mental health” OR psychology OR “psychological response” OR psychophysiological OR “psychophysiological response”) AND (“physical activity” OR “physically active” OR athletes)

**Databases used**	Web of Science, PubMed/MEDLINE, Scopus, SPORTDiscus, Embase, ProQuest Central, Cochrane Library, PsycInfo, and SCIELO

**Inclusion criteria**	P (participant): Physically active individuals (i.e., those who complete at least 150 to 300 min moderateintensity activity or 75–150 min of vigorous-intensity activity a week for health, fitness, or recreational purposes) and athletes (i.e., individuals who train regularly at least three times per week with the purpose to compete) I (intervention): Diurnal sleep or napping implemented following normal sleep (i.e., 7–9 hours) or partial sleep deprivation C (comparison): No-nap condition following the same sleep night O (outcome): Psycho-physiological measures S (study design): Experimental studies (e.g., randomised, nonrandomised trials, interventional trials)

**Exclusion criteria**	P: Individuals who do not practice any kind of physical activity. I: Daytime napping implemented during Ramadan fasting C: None O: Outcomes not described in sufficient details S: Expert opinions, commentaries, conference abstracts or proceedings, reviews, letters to the editor, editorials

**Time filter**	No restrictions were applied in terms of time frame

**Language filter**	No restrictions were applied in terms of language

**Hand-searched target journals**	British Journal of Sports Medicine, Journal of Sports Sciences, International Journal of Sport Physiology and Performance, International Journal of Environmental Research and Public Health, Sports, European Journal of Sport Sciences, Sleep Medicine, Sleep, Chronobiology International, Biological Rhythm Research, Journal of Sleep Research, Asian Journal of Sports Medicine

### The eligibility criteria

The PICO(S) strategy was followed in the current study. The inclusion criteria for this review focused on studies involving physically active individuals and athletes. The intervention of interest is diurnal sleep or napping conducted after a normal night’s sleep (7–9 hours) or following partial sleep deprivation. Studies must include a no-nap condition as a comparison, maintaining the same prior sleep conditions. The primary outcomes assessed are psycho-physiological measures. However, studies are excluded if they involve individuals who do not engage in any form of physical activity. Additionally, studies examining daytime napping during Ramadan fasting are excluded because the observance of Ramadan may affect sleep-wake patterns in athletes and physically active individuals, potentially confounding the effects of napping. Studies are also excluded if outcomes are not described in sufficient detail. Finally, expert opinions, commentaries, conference abstracts or proceedings, reviews, letters to the editor, and editorials are excluded. All the search criteria are summarised in Table 1.

### Study selection and data collection

**FIG. 1:**
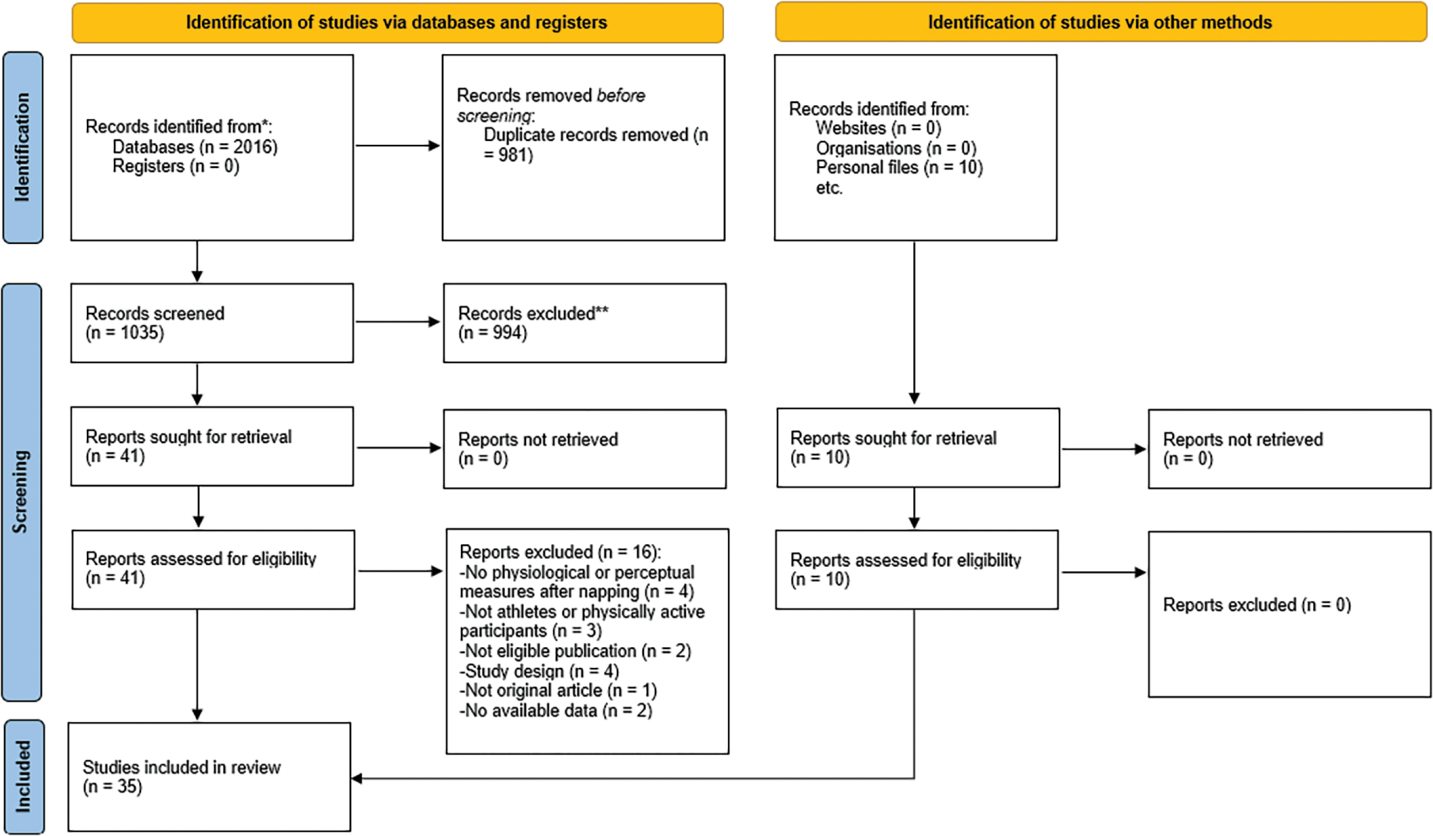
PRISMA flow diagram.

A summary of the selection procedure is presented in Figure 1. EndNote 21 was used to eliminate duplicate articles recorded in the initial search. Two independent authors (OB and KT) checked the eligibility of the included studies, and any disagreements were resolved by a third reviewer (MD). First, the authors checked the eligibility of the articles by screening titles and abstracts. Then, the selected eligible articles were fully checked. Finally, all studies that met the inclusion criteria were included.

The following data were extracted from the included articles by two authors (OB and KT), and disagreements were resolved by consensus: participant characteristics (number of participants, age, level of practice, and activity), study characteristics (nap duration, time of day of napping, time between the end of napping and the exercise), and key findings.

### Risk of bias assessment

Two authors (OB and KT) performed a quality assessment of each study using the Cochrane Collaboration’s risk of bias tool (RoB 2.0) [29]. The RoB 2 tool assesses risk of bias across five key domains: “randomization process”, “deviations from intended interventions”, “missing outcome data”, “measurement of the outcome”, and “selection of the reported result”. A trial was judged as “low risk” only if all five domains were rated as low risk. A rating of “some concerns” was given if one or more domains were judged as having “some concerns,” while a “high risk” rating was applied if at least one domain was judged as “high risk”. This assessment allowed us to systematically evaluate the methodological rigor of the included studies and determine the potential influence of bias on the reported findings.

### Certainty of evidence assessment

To rate the certainty of the evidence (CoE) provided by this review, the Cochrane Grading of Recommendations Assessment, Development and Evaluation (GRADE) approach was employed [30]. This evaluation focused on 10 outcomes analyzed in the meta-analyses. GRADE assessment involves examining the methodological flaws of the studies (i.e., risk of bias), the heterogeneity of results across studies (i.e., inconsistency), the generalizability of the findings to the target population (i.e., indirectness), imprecision of estimates and the risk of publication bias [30].

### Meta-analysis

Analyses were conducted using R (Version 4.3.1; R Core Team, 2021) and the R package “metafor” (Version 4.2.0; [31]). A significance level of p < .05 was set for all analyses.

A random-effects model based on the DerSimonian-Laird approach [32] was initially employed to pool data for psychophysiological measures in each no-nap (CON) and nap condition. Mean values for psychophysiological measures, with their corresponding 95% confidence intervals (CIs), were reported.

To address dependency issues from including multiple data points from the same studies, a three-level meta-analysis (multilevel model) was used [33, 34]. This approach separates variance components into sampling variance (level 1), within-study variance (level 2), and between-study variance (level 3). Forest plots illustrated point estimates of the weighted effect sizes (ESs) and their 95% CIs.

The standardized mean difference (SMD) was used as the measure of ES, with values of 0.8 indicating a large effect, 0.5 a moderate effect, and ≤ 0.2 a small effect [35]. A negative ES value indicates that the results favor the CON, while a positive ES favors napping.

Statistical heterogeneity was assessed using Q [36] and I^2^ statistics [37]. Moderator analysis was performed through subgroup analysis for categorical variables (i.e., level of practice, activity type, time of day of napping, exercise type, scale type) and meta-regression for continuous variables (i.e., age, nap duration, time between the end of napping and testing).

### Outlier Detection

Diagnostics for leverage, outliers, and influential cases were conducted using hat values, Cook’s distance, and studentized residuals. [31, 38, 39] Cases were flagged if their hat values and Cook’s distance exceeded three times the mean, with studentized residuals greater than 3 in absolute terms.

### Publication bias

To assess potential publication bias, we evaluated funnel plot asymmetries, applied Begg and Mazumdar’s rank correlation test [40], and the multilevel model of Egger’s test [41].

### Protocol deviations

The primary deviation from our protocol was the use of the RoB 2 tool for randomised trials to assess the risk of bias in randomised studies, instead of the QUALSYST tool [42], which is typically applied across all study designs.

## RESULTS

### Study selection

The initial search yielded 2016 individual records, of which 1035 remained after removing duplicates. Subsequently, 41 published articles were retained after reviewing the titles and abstracts (Figure 1). After a thorough examination of the 41 full texts, 25 articles were deemed relevant and included. 10 additional articles were identified through the review of reference lists and related citations found via Google Scholar, resulting in a total of 35 articles. Details on the search strategy results used are provided in Table S2. The reasons for excluding articles during the full-text screening stage are outlined in Table S3.

### Study characteristics

A total of 35 studies [11, 17–23, 43–69] which involved 489 participants, were included in this meta-analysis. These studies were published between 2007 and 2024. The characteristics of the included studies can be found in Table S4. Among these studies, the one conducted by Pelka et al. [44] had the largest number of participants, which was 27. The number of participants in the other studies ranged from seven to 27. The average age of the participants varied between 15 and 35 years. Of all the studies reviewed, only O’Donnell et al. [20] and Willmer et al. [67] included female athletes. The remaining studies focused exclusively on male participants. The study population consisted of physically active participants who were moderately trained in seven studies and trained athletes in 27 studies. The included studies examined the acute effects of daytime napping on psychophysiological measures following NS or partial SD (PSD).

### Results from meta-analyses

#### Effect of napping on psychophysiological measures

##### Normal sleep

**FIG. 2:**
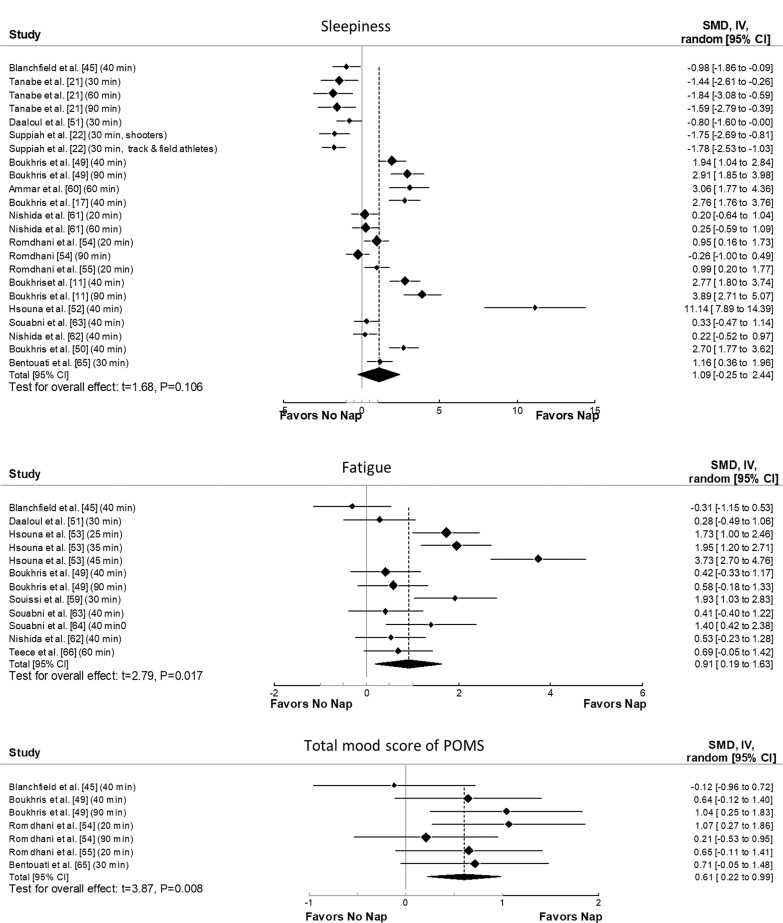
Forest plot of the impacts of napping after normal sleep on sleepiness, fatigue, and mood states. SMD: standardized mean difference, CI: confidence intervals.

**FIG. 3:**
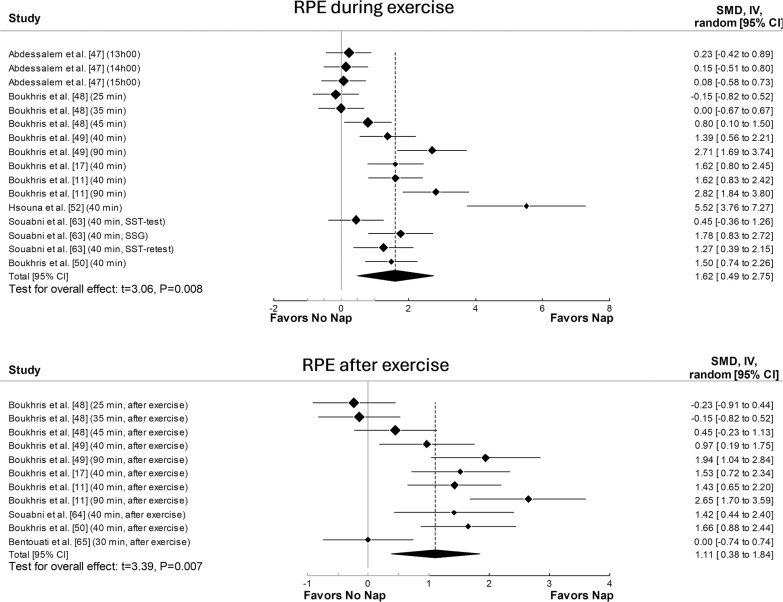
Forest plot for the impacts of napping following normal sleep on rating of perceived exertion (RPE) during and after exercise. SMD: standardized mean difference, CI: confidence intervals.

**FIG. 4:**
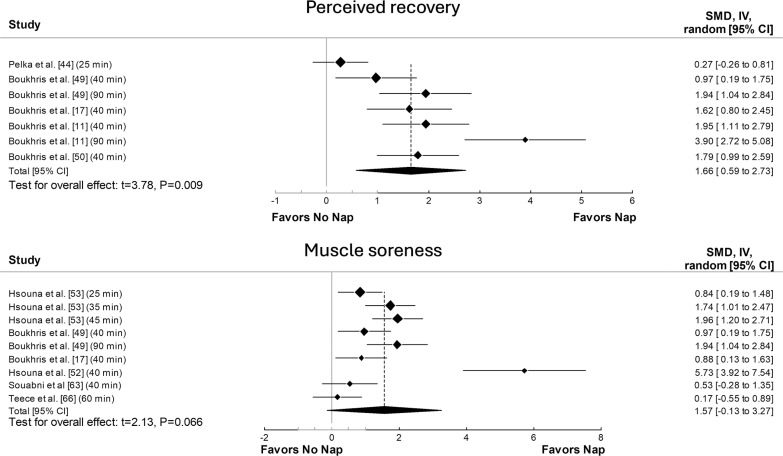
Forest plot for the impacts of napping following normal sleep on perceived recovery and muscle soreness. SMD: standardized mean difference, CI: confidence intervals.

**FIG. 5:**
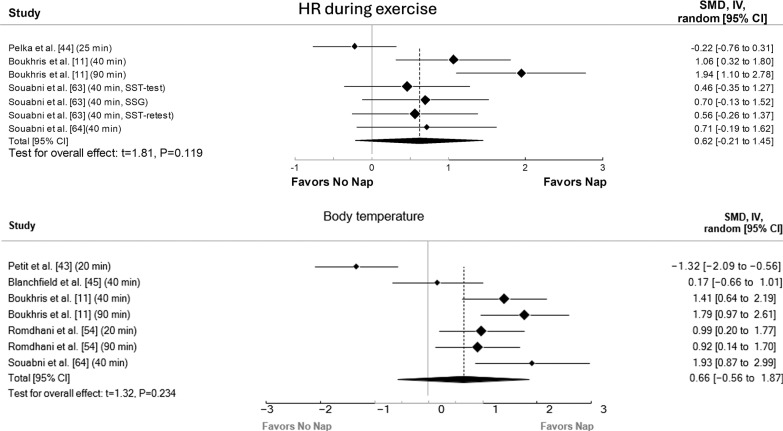
Forest plot for the impacts of napping following normal sleep on heart rate (HR) during exercise and body temperature. SMD: standardized mean difference, CI: confidence intervals.

The principal analysis indicated that taking a nap reduced total mood score of the Profile of Mood States (POMS) (SMD = 0.61, p = 0.008, Figure 2), fatigue (SMD = 0.91, p = 0.01, Figure 2), RPE (Figure 3) both during (SMD = 1.62, p = 0.008) and immediately after exercise (SMD = 1.11, p = 0.007). Additionally, taking a nap improved perceived recovery (SMD = 1.66, p = 0.009, Figure 4). However, there were no significant effects of napping on sleepiness (SMD = 1.09, p = 0.10, Figure 2), muscle soreness (SMD = 1.57, p = 0.06, Figure 4), HR during exercise (SMD = 0.62, p = 0.11, Figure 5), and temperature (SMD = 0.66, p = 0.23, Figure 5).

Significant heterogeneity was observed across most outcomes (i.e., sleepiness, fatigue, RPE both during and after exercise, perceived recovery, muscle soreness, HR, and body temperature). The only exception was total mood score of POMS, which showed no significant heterogeneity. The heterogeneity statistics for each outcome are summarized in Table S5.

Meta-regressions showed that nap duration had a significant moderating effect on RPE both during (F_(1, 14)_ = 10.82, p = 0.005) and after (F_(1, 9)_ = 16.22, p = 0.003) exercise, with each 1-minute increase in nap duration leading to an increase in the ES by 0.01 and 0.026, respectively.

Subgroup analysis revealed a significant moderating effect of the level of practice on RPE during exercise (F_(1, 14)_ = 5.51, p = 0.03), with athletes showing a larger effect (ES = 2.09, p = 0.003) compared to physically active individuals (ES = 0.18, p = 0.23). Additionally, activity type moderated RPE during (F(_1, 14)_ = 5.51, p = 0.03) and after exercise (F_(2, 8)_ = 37.8, p < 0.001), with team sports having a greater impact both during (ES = 2.09, p = 0.003) and after exercise (ES = 1.62, p < 0.001) compared to individual sports (during: ES = 0.18, p = 0.23; after: ES = 0.02, p = 0.93). A significant effect of the measuring tool on sleepiness was observed (F_(3, 19)_ = 3.4, p = 0.03), with the Stanford Sleepiness Scale (ES = 3.16) showing a greater effect than the Epworth Sleepiness Scale (ES = -1.43). Finally, the level of practice significantly moderated fatigue (F_(1, 10)_ = 28.3, p < 0.001), where physically active individuals had a larger effect (ES = 2.28) compared to athletes (ES = 0.39).

### Partial sleep deprivation

The principal analysis indicated that the effects of napping on sleepiness (SMD = 1.03 (large), p = 0.11) and fatigue (SMD = 0.79 (moderate), p = 0.07) were not significant (Figure S1).

Significant heterogeneity was observed in sleepiness and fatigue (see Table S5 for details). However, meta-regression and subgroup analyses did not find any significant moderating effects on fatigue, and were not conducted for sleepiness due to the limited number of studies.

### Publication bias

Publication bias was observed for the effects of naps following normal sleep on RPE during and after exercise, perceived recovery, and muscle soreness, as well as for sleepiness following PSD (more details in the supplementary file (Figure S2–12)).

### Results without meta-analyses

#### Napping following normal sleep

##### Psychological measures

Four studies investigated the impact that napping has on vigor [45, 49, 59, 64], with two studies finding no significant impact from a 30-min nap [59] or a 40-min nap [45]. However, a significant increase in vigor was observed in the remaining two studies after a 40-min nap [50, 64] and a 90-min nap [49].

Three studies assessed how napping affects stress [44, 53, 63]. Daytime naps of 25-, 35-, and 45-min lowered stress, with the 45-min nap being most effective [53]. Similarly, a 40-min nap significantly reduced stress compared to CON [63]. However, Pelka et al. [44] found no significant impact from a 25-min nap on stress.

Three studies investigated how napping impacts depression and confusion [49, 59, 64]. A 40-min nap showed no significant effect on either measure in one study [64]. However, a 30-min nap significantly decreased both depression and confusion compared to CON [59]. Boukhris et al. [49] found no significant impact on confusion but did observe a significant decrease in depression after both a 40-min and a 90-min nap, with the longer nap being more effective.

Two studies each examined the effects of napping on tension [49, 59] and anger [49, 64]. Boukhris et al. [49] found that both a 40-min and a 90-min nap significantly reduced both tension and anger, with greater improvement after the 90-min nap. Similarly, Souissi et al. [59] reported a significant decrease in tension after a 30-min nap compared to CON. However, a 40-min nap did not significantly impact anger levels in one study [64].

One study found that a 40-min nap significantly reduced anxiety compared to CON [64].

Two studies investigating the effects of napping on the Hooper index found no significant effect [55, 63].

Two studies found no significant effect of napping on some subjective measures [20, 53]. O’Donnell et al. [20] found no impact on perceived performance or energy level, while Hsouna et al. [53] observed no changes in feelings scores.

### Physiological measures

Four studies examined napping’s effect on lactate levels during/after exercise [43, 54, 56, 59]. No significant impact was found after a 20-min [43] or 30-min nap [59]. However, lactate increased significantly after a 20-min nap [54, 56], with a significant decrease observed after a 90-min nap compared to a 20-min nap [54].

Two studies examined napping’s effect on lactate dehydrogenase (LDH) levels [17, 54]. Boukhris et al. [17] found a 40-min nap was associated with decreased LDH levels before and after exercise compared to CON. Romdhani et al. [54] found no effect from a 20-min nap, but a 90-min nap was associated with decreased post-exercise LDH compared to both CON and a 20-min nap.

Two studies examined napping’s effect on creatine kinase (CK) and aspartate aminotransferase (ASAT) levels [17, 56]. Boukhris et al. [17] found a 40-min nap was associated with decreased CK and ASAT levels before and after exercise compared to CON. However, Romdhani et al. [56] found no significant effect from a 20-min nap on either CK or ASAT levels.

One study investigated napping’s effect on alanine aminotransferase (ALAT) and C-reactive protein (CRP) levels [17]. The findings revealed that a 40-min nap was associated with reduced ALAT and CRP levels before and after exercise compared to CON.

Two studies examined napping’s effect on glycemia, glutathione peroxidase (GPx), and superoxide dismutase (SOD) levels [54, 56]. Romdhani et al. [54] found a 20-min nap was associated with increased glycemia, GPx, and SOD levels compared to CON, while a 90-min nap was associated with increased glycemia and SOD. However, Romdhani et al. [56] found a 20-min nap was associated with increased SOD, with no significant effect on glycemia or GPx.

Two studies have examined the effect of napping on urea [54, 56]. The authors found that napping did not increase urea postexercise; however, urea increased significantly postexercise in the CON [54, 56].

Blanchfield et al. [45] did not find any significant effect of a 40-min nap on urine specific gravity.

Two studies found a 40-min nap decreased HRpeak compared to CON [63, 64].

Two studies examined the effect of napping on HR variability (HRV) [11, 22]. Suppiah et al. [22] did not find any significant effect of a 30-min nap on the mean R–R interval (RR), root mean square of successive R–R intervals (RMSSD), high frequency (HF), low frequency (LF), or LF/HF. However, Boukhris et al. [11] reported that the HRV score, RR, RMSSD, percentage of RR intervals with a difference in duration greater than 50 ms (PNN50), standard deviation of normal-to-normal intervals (SDNN), HF, LF, and LF/HF improved significantly after both a 40-min nap and a 90-min nap, with a greater improvement observed after a 90-min nap than after a 40-min nap.

One study examined napping’s impact on blood pressure, finding significant decreases in both diastolic and systolic blood pressure after both a 40-min and 90-min nap, with greater decreases after the longer nap [11].

One study examined the effect of 25-min and 45-min naps on respiratory function, finding significant increases in peak expiratory flow values only after the 45-min nap compared to both CON and a 25-min nap [68].

One study examined the effect of a 40-minute nap on neuromuscular fatigue, revealing that neuromuscular responses were positively affected by the nap [50].

### Napping following partial sleep deprivation

#### Psychological measures

Four studies investigated the impact of napping on vigor [19, 58, 59, 69]. Increased vigor was found after a 30-min nap [59] and a 60-min nap [19]. However, Romdhani et al. [58] found no significant effect on vigor after either a 20-min or 90-min nap. Similarly, Gallagher et al. [69] found no significant effect on vigor after either a 30-min or 60-min nap.

Four studies investigated napping’s effects on depression and confusion [19, 58, 59, 69]. Decreases in both were observed after a 30-min [59] and a 60-min nap [19]. However, Romdhani et al. [58] found confusion unchanged after both a 20-min and a 90-min nap compared to CON. They did find depression significantly decreased only after a 90-min nap. Gallagher et al. [69] found no significant effect on depression and confusion after either a 30-min or 60-min nap.

Three studies investigated napping’s effect on tension [19, 58, 59, 69]. Naps of 20-min [58], 30-min [69], 60-min [19, 69], and 90-min [58] showed no significant effect. However, Souissi et al. [59] found a 30-min nap significantly decreased tension compared to CON.

Three studies examined napping’s effect on anger [19, 58, 69]. A 60-min nap significantly decreased anger compared to CON [19]. However, Romdhani et al. [58] found anger significantly decreased after a 90-min nap compared to a 20-min nap. In contrast, Gallagher et al. [69] found no significant effect on anger after either a 30-min or 60-min nap.

Two studies examined napping’s effect on happiness and calmness [19, 69]. A 60-min nap significantly increased both [19]. However, Gallagher et al. [69] found no significant effect on happiness and calmness after either a 30-min or 60-min nap.

One study investigated the impact of a 20-min nap on total mood score and found no significant effect [55].

Two studies examined napping’s effects on the Hooper index [55, 58]. Both found significant decreases after 20-min and 90-min naps, with greater decreases after the longer nap [58].

### Physiological measures

Three studies examined the effect of napping on lactate [57–59]. Romdhani et al. [57], [58] found post-exercise lactate significantly increased after a 20-min nap compared to CON, with no significant changes after a 90-min nap. Conversely, Souissi et al. [59] found lactate measured immediately after exercise decreased significantly after a 30-min nap compared to CON.

Two studies examined the effect of napping on temperature [19, 69]. Gallagher et al. [69] found a significant effect after both 30-min and 60-min naps. However, Brotherton et al. [19] found no significant impact from a 30-min nap.

Two studies examining the effect of napping on LDH found no significant impact from either a 20-min or a 90-min nap [46, 57].

Only one study examined the effect of napping on white blood cells (WBC), red blood cells (RBC), mean corpuscular volume (MCV), mean corpuscular hemoglobin (MCH), mean platelet volume (MPV), magnesium ions (MG^++)^, platelets (PL), lymphocytes (LY), monocytes (MO), hematocrit (HT), hemoglobin (HB), sodium (NA^+^), potassium (K^+^), and ALAT [46]. The authors did not find any significant effect of either a 20-min nap or a 90-min nap on the WBC or RBC [46]. However, MCH and MG^++^ were greater after a 20-min nap than after a CON and lower after a 90-min nap than after a 20-min nap [46]. On the other hand, the MCV was lower after a 20-min nap than after a CON, with a greater decrease after a 90-min nap than after a 20-min nap [46]. LY, MPV and MO were significantly greater after both a 20-min nap and a 90-min nap than after CON, with greater increases in MPV and MO observed after a 90-min nap than after a 20-min nap [46]. HT, HB, NA^+^, K^+^, and ALAT did not change after a 20-min nap; however, they significantly increased after a 90-min nap compared to CON, with greater increases in HB, NA^+^, and K^+^ after a 90-min nap compared to a 20-min nap [46]. Nevertheless, the PL increased after a 20-min nap compared to CON [46].

Two studies examined the effect of napping on glycemia, CK, SOD, UA, ASAT, and GPx [57, 58]. The authors did not find any significant effect of either a 20-min nap or a 90-min nap on CK or glycemia [57, 58]. With respect to SOD, Romdhani et al. [57] reported an increase after a 20-min nap compared to CON. However, Romdhani et al. [58] did not find any significant changes. For UA, there was a significant increase after both a 20-min [57, 58] and a 90-min [58] nap compared to CON. On the other hand, for ASAT, there was a significant decrease after both a 20-min [57, 58] and a 90-min [58] nap compared to CON. Regarding GPx, Romdhani et al. [57] reported an increase after a 20-min nap compared to CON. However, Romdhani et al. [58] reported an increase after a 90-min nap compared to CON and a 20-min nap.

One study on urea levels found a significant decrease after a 90-min nap compared to both CON and a 20-min nap [58]. Similarly, a single study examining brain wave activity found no significant effect from a 20-min nap [23].

### Risk of bias assessment

The overall risk of bias evaluation revealed that 86% of the studies had some concerns, while 14% were rated as high risk. A detailed report of the risk of bias assessment can be found in Table S6.

### Certainty of evidence

Due to the experimental design nature of the included studies, the GRADE assessment started with a low CoE. The GRADE results indicate very low CoE for mood state, body temperature, and heart rate (HR) during exercise following normal sleep, as well as fatigue following PSD, due to serious risk of bias and very serious imprecision. However, CoE for sleepiness, fatigue, perceived recovery, and muscle soreness following NS, as well as sleepiness following PSD, was rated as low due to imprecision (i.e., number of participants < 400 and wide CI) and risk of bias, with an upgrade applied based on the large SMD observed. In contrast, CoE for rating of perceived exertion (RPE) during and after exercise was rated as moderate due to imprecision and risk of bias, with an upgrade based on the large SMD and dose-response relationship. Details of the assessment are presented in Table S7.

## DISCUSSION

This review is the first systematic review and meta-analysis summarizing the impacts of diurnal napping following NS and PSD on psychophysiological measures in athletes and physically active individuals. Our analysis revealed that during NS, daytime napping taken in the afternoon (13:00–16:00) had a positive influence on various psychological indicators, including fatigue, mood state, RPE during and immediately after exercise, and perceived recovery. Regarding physiological measures, our results indicated no significant impact of napping on HR during exercise and post-nap body temperature. When considering PSD, the present findings showed that diurnal napping did not alleviate sleepiness and fatigue. The overall CoE was rated as ‘moderate’ for RPE during and after exercise following NS. However, the CoE for all other outcomes was classified as low to very low, indicating that these findings should be interpreted with caution. However, due to the limited number of available studies, we were unable to draw definitive conclusions about the remaining perceptual and physiological measures after NS or PSD.

### Napping following normal sleep

#### Psychological measures

The results of the meta-analysis showed that daytime napping after NS improved perceptual measures, including fatigue, perceived recovery, RPE during and immediately after exercise, and muscle soreness.

Although our meta-analysis showed a large ES for napping on sleepiness (SMD = 1.09), the effect was not significant (p = 0.10). This may be due to many studies measuring sleepiness immediately after waking, when sleep inertia is most prominent [21, 45]. This immediate assessment might underestimate the true benefit of napping on subsequent sleepiness, as the effects of sleep inertia could impact negatively the results. Future research should assess sleepiness over a longer post-nap period, allowing sleep inertia to dissipate. Subgroup analysis also revealed a significant moderating effect of the measurement tool on sleepiness, with sleepiness measured using the Stanford Sleepiness Scale (ES = 3.16) showing a large effect compared to sleepiness measured using the Epworth Sleepiness Scale (ES = -1.43). This discrepancy highlights the importance of tool selection and suggests the Stanford Sleepiness Scale might be more sensitive to changes in subjective sleepiness following a short nap, whereas the Epworth Sleepiness Scale is designed to assess habitual sleepiness, which might not capture the immediate, short-term effects of napping. Standardising assessment tools in future studies would improve comparability. Significant heterogeneity (I^2^ = 96.6%) suggests variation in study designs and nap protocols, further emphasizing the need for more consistent methodologies.

Our meta-analysis pooling revealed that napping resulted in a significant decrease in fatigue and total mood score, and a significant increase in perceived recovery compared to CON. These findings show that napping could help the body recover from physical and mental exertion, which could be explained by the potential of napping to facilitate neural and peripheral cellular restoration, improve energy conservation, and reduce inflammation levels [7, 17]. Subgroup analysis indicated that physically active individuals experienced a greater reduction in fatigue than athletes, potentially due to differences in training intensities. This is consistent with previous findings, showing napping’s benefits in physical and cognitive performance, as well as perceived fatigue, are more pronounced in physically active individuals [12]. However, meta-regressions and additional subgroup analyses were not conducted on perceived recovery, and mood states due to the small number of studies, preventing us from fully explaining the observed heterogeneity. Further research is needed to address these gaps.

The meta-analytic pooling of RPE data revealed significant decreases in perceived exertion when napping compared to CON, both during and after exercise. Meta-regression and subgroup analyses indicated that RPE may be influenced by two key factors: the type of activity (team vs. individual sport) and nap duration. Results showed a greater reduction in RPE in team sports compared to individual sports, with longer naps contributing more to this effect. The collaborative nature of team sports may lead to greater psychological stress and pressure [70], which could explain why athletes in team sports benefit more from napping, as the recovery period may help alleviate both physical and mental fatigue. Additionally, a longer nap that includes more time in deeper sleep stages may be required to mitigate the perception of fatigue and physical and mental exhaustion resulting from the sport activity [10, 11]. However, it remains unclear why team sport athletes perceive less exertion compared to individual athletes, and future studies are needed to clarify this finding.

Although our meta-analysis showed a large ES for napping on muscle soreness (SMD = 1.57), the effect was not significant. This finding is particularly interesting considering that among the six studies included in the analysis [17, 49, 52, 53, 63, 66], only two did not report a significant effect of napping on muscle soreness [63, 66]. The first study [53] that did not find a significant effect may be attributed to the absence of sleep measurement during the nap itself. Without proper assessment of sleep quality and duration, the impact of napping on muscle soreness may not be accurately captured. Regarding the second study [66], only 30 minutes were allowed after waking from the nap to avoid sleep inertia, which recent meta-analyses suggest is insufficient for achieving the desired recovery benefits [9, 12]. Additionally, with a total of only 88 participants across all studies examining muscle soreness, sample size and power issues may also have contributed to the lack of significance in our findings. It is important to note that meta-regression and subgroup analyses were not conducted for muscle soreness due to the limited number of available studies. Such analyses could have helped identify potential moderating effects of various factors, such as nap duration, timing, and participant characteristics. Despite the lack of statistical significance, the large ES suggests that napping may still have practical relevance for muscle recovery. Athletes and coaches should consider individual variability, as well as factors such as nap duration and timing, when integrating naps into recovery strategies. Therefore, further studies with robust methodological designs are required to explore these aspects and provide a clearer understanding of the relationship between napping and muscle soreness.

Although we did not conduct a meta-analysis on some perceptual measures due to the limited number of studies, napping had favorable effects on multiple variables: two out of four studies supported the positive impact of naps on vigor, two out of three studies supported its effect on stress and depression, one out of three studies supported its effect on confusion, two out of two studies supported its impact on tension, one out of two studies supported its effect on anger, and one out of one study supported its effect on anxiety. However, no studies among the available studies favor napping for the Hooper index, perceived performance, energy levels, or feelings score. Further studies on these perceptual measures are required to draw firm conclusions.

### Physiological measures

The present findings showed that daytime napping following NS did not significantly affect HR during exercise and post-nap body temperature. However, the low number of studies on the remaining physiological measures precluded drawing firm conclusions on these outcomes.

While the overall ESs suggested potential benefits of napping on HR during exercise and body temperature, the meta-analysis revealed no statistically significant differences compared to the CON condition. This apparently contradictory finding, moderate to large ESs but non-statistically significant results, highlights the limitations of the available data and strongly suggests insufficient statistical power due to the small number of included studies. In fact, comprehensive meta-regressions and subgroup analyses were not conducted due to the restricted small number of studies included in the analyses for these outcomes. Consequently, we could not reliably investigate potential moderators that might explain the observed heterogeneity between studies and clarify the true influence of napping on these physiological variables. Several unexplored factors, such as nap timing and duration, and individual differences in training status and age, could influence the physiological responses to napping. Future research should prioritize investigating these moderators using adequately powered studies with sufficient sample sizes to conduct meaningful subgroup analyses to provide a clearer understanding of how napping interacts with physiological responses. Such studies could offer insights into optimizing nap protocols for athletes and physically active individuals, potentially enhancing recovery strategies and overall performance.

Due to the limited number of studies examining lactate, glycemia, GPx, SOD, urea, urine specific gravity, HRV, blood pressure, muscle damage, and inflammation, a meta-analysis was not conducted. However, some existing studies suggest napping may benefit these physiological measures. For instance, daytime naps have been shown to reduce muscle damage and inflammation caused by intense exercise [17, 54], potentially delaying fatigue and enhancing performance. Additionally, napping could attenuate the rise in urea levels associated with exercise [54, 56], which may protect both the brain and muscles from the detrimental effects of increased ammonia [54]. Napping also impacts autonomic function, as evidenced by changes in HRV and blood pressure, possibly due to its influence on cardiac function [11]. By increasing parasympathetic activity, napping may reduce sympathetic hyperactivity and pro-inflammatory cytokines, improving cardiovascular regulation and facilitating recovery, which is crucial for athletic performance [11].

### Napping following partial sleep deprivation

#### Psychological measures

Limited research on daytime napping following PSD allowed for a meta-analysis focusing primarily on its effects on sleepiness and fatigue. Although the results revealed that napping did not significantly affect either sleepiness or fatigue, it is important to note that the effect sizes were large for sleepiness and moderate for fatigue. These findings suggest that napping has the potential to alleviate the negative effects of sleep deprivation, even if statistical significance was not achieved, potentially due to limitations in the data and variability across studies.

The variability in SD protocols, such as differences in duration and severity of deprivation, as well as inconsistencies in nap characteristics such as timing and length, may have contributed to the lack of significant findings. For example, shorter naps or naps taken too soon after waking may not fully mitigate sleep inertia, while longer naps or naps later in the day may be more effective in reducing sleepiness and fatigue. This inconsistency between studies could explain why, despite the observed large effect size, napping’s impact on sleepiness did not reach statistical significance.

Additionally, previous research has shown that napping consistently reduces sleepiness and improves mood in sleep-deprived individuals [18, 19, 55, 58], suggesting that even moderate reductions in fatigue might be linked to improvements in mood and overall cognitive function. These promising results highlight the need for future studies to standardize SD protocols and nap conditions to better understand the potential benefits of napping in counteracting SD.

### Physiological measures

A limited number of studies have examined the impact of napping on physiological responses following PSD. While some results are conflicting, studies suggested potential benefits. For example, napping, compared to CON, has been linked to decreased HR, possibly due to increased parasympathetic activity [18]. However, a short 20-minute nap did not affect brain wave activity, potentially because this duration is insufficient for deeper stages of sleep [23].

Regarding blood lactate during/after exercise, while some studies found increases after short naps (20–30 minutes), a 90-min nap resulted in no change compared to no nap [55, 58, 59]. This discrepancy might be attributed to the longer nap’s greater aerobic contribution to energy synthesis and its association with lower body temperature [58].

Two studies observed lower body temperature after napping following PSD [18, 58], and one found no effect [19]. This difference might be attributed to SD severity across studies, as Brotherton et al. [19] involved two nights of PSD, whereas the other two studies had only one night [18, 58].

Additionally, napping may improve oxidative damage by eliminating reactive oxygen species [58]. A longer 90-min nap also enhanced hematological and biochemical responses to exercise more than shorter naps, potentially because the greater increase in physical performance after the 90-min nap led to higher hematological and biochemical demands [46].

### Methodological considerations

Napping studies often lack accurate sleep measurement as most studies haven’t used polysomnography, the gold standard. Instead, most estimated nap duration [18–20, 44, 46–48, 53–60, 65, 68, 69] or used basic tools, such as actigraphy [11, 17, 45, 49–52, 63, 64], which don’t provide detailed sleep stage information. This lack of detail limits understanding of napping benefits for athletes. Future studies need sensitive tools, ideally polysomnography or similar advancements allowing natural sleep at home, to objectively measure sleep stages during naps.

The current evidence suggests that napping could benefit recovery, but the optimal duration for maximum benefits remains unclear. Based on the analysed data, naps ranging from 25 to 90 minutes appear to offer the most practical and effective benefits for athletes and physically active individuals, particularly when taken in the afternoon (13:00–16:00). Longer naps (e.g., 90 minutes) may offer more profound recovery by allowing for more sleep time and deeper sleep stages. However, the benefits of longer naps may be moderated by individual factors, such as chronotype or habitual napping patterns, and potentially weighed against the impact on nocturnal sleep. Further research is required to define the ideal duration more precisely.

An important consideration when assessing the effectiveness of napping is the role of individual variability. Athletes may vary widely in their baseline sleep needs, prior sleep debt, extended wake duration, training loads, and recovery capabilities. Factors such as chronotype (morning vs. evening preferences), habitual napping, and personal stress levels could influence how an individual responds to napping. For example, individuals with different chronotypes may experience enhanced recovery if naps are timed in alignment with their natural circadian rhythms [71, 72]. Indeed, a ‘lark’ (morningtype) may benefit from early afternoon naps when their energy dips, while an ‘owl’ (evening-type) may find late afternoon or early evening naps more restorative, aligning with their later peak alertness. These individual differences highlight the need for personalised nap strategies that consider not just the duration and timing of naps but also the individual’s lifestyle and body clock.

It is essential to consider sleep inertia when scheduling daytime naps. While no studies confirm the exact time needed to counteract its negative effects on athletes, research on inactive individuals [9] shows performance impairments may last up to 2 hours after waking. Studies in this review allowed participants 30 to 270 minutes to overcome sleep inertia. Future research should examine sleep inertia’s effect on athletic performance and strategies to mitigate it.

Daytime naps can impact nighttime sleep, particularly sleep onset latency. Factors such as SD, nap length, and timing all play a role. One study found naps, especially in older adults, may increase sleep onset time [73]. However, further research is needed to confirm this link and explore ways to mitigate sleep disruption from daytime napping.

The certainty of evidence assessment, following the GRADE approach, revealed varying levels of confidence in the effect of napping on psychophysiological measures. While all studies employed randomised crossover design, which initially suggests a low certainty of evidence according to the GRADE system, when assessing certainty. The assessment considered factors that could enhance this certainty, particularly the presence of large SMD and dose-response relationship. Notably, imprecision due to small sample sizes and wide CI was a consistent concern across all outcomes. Moderate certainty of evidence was found only for the beneficial effects of napping on RPE during and after exercise. This assessment was supported by the large effect sizes observed and a clear dose-response relationship, as revealed by meta-regression. The evidence for sleepiness, fatigue, perceived recovery, and muscle soreness following normal sleep, as well as sleepiness following sleep deprivation, remained at a low certainty level. Furthermore, the evidence supporting effects on mood states, body temperature, and HR during exercise following NS, as well as fatigue following PSD, was rated as very low. These findings highlight the need for future research to prioritize larger sample sizes and standardised methodologies to address the issue of imprecision and enhance the certainty of evidence across all outcomes.

Future napping studies should adopt standardised methodologies by using polysomnography or advanced wearables to measure sleep stages accurately and schedule naps during the early afternoon (13:00–16:00) to align with circadian rhythms. Studies should compare short naps (25–40 minutes) with longer naps (60–90 minutes), always including a no-nap control. Post-nap testing should occur at least one hour after waking to avoid the potential effects of sleep inertia. Individual factors such as chronotype and habitual napping should be assessed to explore variability in nap effectiveness. The impact of napping on nighttime sleep should also be evaluated using actigraphy or polysomnography. Finally, studies should prioritize larger, diverse samples, and longitudinal designs, to provide practical recommendations for athletes.

### Strengths and weaknesses of the review

This is the first systematic review and meta-analysis to examine the impacts of diurnal napping on psychophysiological measures in athletes and physically active individuals, considering both NS and PSD. It offers comprehensive coverage of the literature and a careful quality appraisal. We conducted a thorough search across nine databases without time or language restrictions. Importantly, this is the first review to assess CoE in a systematic review on napping, despite it being a mandatory requirement with PRISMA 2020 guidelines [26]. The high heterogeneity of the included studies is a limitation, which was addressed through meta-regression and subgroup analyses. However, the results of the meta-regression and subgroup analyses should be interpreted with caution due to their observational nature [74]. Additionally, some parameters were not subjected to meta-analytical calculations due to the small number of studies.

## CONCLUSIONS

This review showed that daytime napping can have positive effects on a range of psychophysiological measures in athletes and active individuals. Napping has been found to have a moderate to large positive impact on fatigue, mood states, perceived exertion, and recovery. However, napping could not help counteract the negative impact of PSD on sleepiness and fatigue. The overall CoE was assessed as ‘moderate’ for RPE during and after exercise following NS. However, the CoE for the remaining outcomes was assessed as low to very low and should be treated with caution. Therefore, future studies with rigorous designs are necessary to enhance the reliability and CoE. Due to the limited number of available studies, further research is required to draw conclusions about the remaining perceptual and physiological measures following NS or PSD.

## Supplementary Material

The effects of daytime napping on psychophysiological measures in physically active individuals and athletes: A systematic review, meta-analysis, and meta-regression, with assessment of the certainty of evidence

## Data Availability

All data and materials are available within the article or supplementary materials: LINK

## Protocol registration

The protocol of this review was registered in the Open Science Framework database (https://doi.org/10.17605/OSF.IO/82KZF).
