# Supplementary material for: The effects of daytime napping on psychophysiological measures in physically active individuals and athletes: A systematic review, meta-analysis, and meta-regression, with assessment of the certainty of evidence
Source: Biol Sport. 2025 Aug 13;43:149–63. doi: 10.5114/biolsport.2026.153310 (PMC12884900; doi:10.5114/biolsport.2026.153310)
Supplement: The effects of daytime napping on psychophysiological measures in physically active individuals and athletes: A systematic review, meta-analysis, and meta-regression, with assessment of the certainty of evidence [file JBS-43-56515-s1.pdf]

Supplementary material to paper:

Boukhris O, Trabelsi K, Suppiah H et al. The effects of daytime napping on psychophysiological measures in physically active individuals and athletes: A systematic review, meta-analysis, and meta-regression, with assessment of the certainty of evidence. Biol Sport. 2026;43(1):149–163. DOI: https://doi.org/10.5114/biolsport.2026.153310

TABLE S1. PRISMA checklist.

Section and Topic	Item #	Checklist item	Location where item is reported
**TITLE**			
Title	1	Identify the report as a systematic review.	1
**ABSTRACT**			
Abstract	2	See the PRISMA 2020 for Abstracts checklist.	3–4
**INTRODUCTION**			
Rationale	3	Describe the rationale for the review in the context of existing knowledge.	5–6
Objectives	4	Provide an explicit statement of the objective(s) or question(s) the review addresses.	5–6
**METHODS**			
Eligibility criteria	5	Specify the inclusion and exclusion criteria for the review and how studies were grouped for the syntheses.	6, Table 1
Information sources	6	Specify all databases, registers, websites, organisations, reference lists and other sources searched or consulted to identify studies. Specify the date when each source was last searched or consulted.	6
Search strategy	7	Present the full search strategies for all databases, registers and websites, including any filters and limits used.	6, Table S2
Selection process	8	Specify the methods used to decide whether a study met the inclusion criteria of the review, including how many reviewers screened each record and each report retrieved, whether they worked independently, and if applicable, details of automation tools used in the process.	7
Data collection process	9	Specify the methods used to collect data from reports, including how many reviewers collected data from each report, whether they worked independently, any processes for obtaining or confirming data from study investigators, and if applicable, details of automation tools used in the process.	7
Data items	10a	List and define all outcomes for which data were sought. Specify whether all results that were compatible with each outcome domain in each study were sought (e.g. for all measures, time points, analyses), and if not, the methods used to decide which results to collect.	7, Table S2
	10b	List and define all other variables for which data were sought (e.g. participant and intervention characteristics, funding sources). Describe any assumptions made about any missing or unclear information.	7, Table S2
Study risk of bias assessment	11	Specify the methods used to assess risk of bias in the included studies, including details of the tool(s) used, how many reviewers assessed each study and whether they worked independently, and if applicable, details of automation tools used in the process.	7
Effect measures	12	Specify for each outcome the effect measure(s) (e.g. risk ratio, mean difference) used in the synthesis or presentation of results.	8–9
Synthesis methods	13a	Describe the processes used to decide which studies were eligible for each synthesis (e.g. tabulating the study intervention characteristics and comparing against the planned groups for each synthesis (item #5)).	8–9
	13b	Describe any methods required to prepare the data for presentation or synthesis, such as handling of missing summary statistics, or data conversions.	8–9
	13c	Describe any methods used to tabulate or visually display results of individual studies and syntheses.	8–9
	13d	Describe any methods used to synthesize results and provide a rationale for the choice(s). If meta-analysis was performed, describe the model(s), method(s) to identify the presence and extent of statistical heterogeneity, and software package(s) used.	8–9
	13e	Describe any methods used to explore possible causes of heterogeneity among study results (e.g. subgroup analysis, meta-regression).	8–9
	13f	Describe any sensitivity analyses conducted to assess robustness of the synthesized results.	8–9
Reporting bias assessment	14	Describe any methods used to assess risk of bias due to missing results in a synthesis (arising from reporting biases).	7

TABLE S1. Continue.

Section and Topic	Item #	Checklist item	Location where item is reported
Certainty assessment	15	Describe any methods used to assess certainty (or confidence) in the body of evidence for an outcome.	7–8
**RESULTS**			
Study selection	16a	Describe the results of the search and selection process, from the number of records identified in the search to the number of studies included in the review, ideally using a flow diagram.	9–10, Figure 1
	16b	Cite studies that might appear to meet the inclusion criteria, but which were excluded, and explain why they were excluded.	Table S3
Study characteristics	17	Cite each included study and present its characteristics.	10, Table S4
Risk of bias in studies	18	Present assessments of risk of bias for each included study.	10, Table S6
Results of individual studies	19	For all outcomes, present, for each study: (a) summary statistics for each group (where appropriate) and (b) an effect estimate and its precision (e.g. confidence/credible interval), ideally using structured tables or plots.	Figure 2, 3, 4, 5 and Figure S1
Results of syntheses	20a	For each synthesis, briefly summarise the characteristics and risk of bias among contributing studies.	12
	20b	Present results of all statistical syntheses conducted. If meta-analysis was done, present for each the summary estimate and its precision (e.g. confidence/credible interval) and measures of statistical heterogeneity. If comparing groups, describe the direction of the effect.	11–12, Table S5
	20c	Present results of all investigations of possible causes of heterogeneity among study results.	11–12
	20d	Present results of all sensitivity analyses conducted to assess the robustness of the synthesized results.	11–12
Reporting biases	21	Present assessments of risk of bias due to missing results (arising from reporting biases) for each synthesis assessed.	Supplementary file
Certainty of evidence	22	Present assessments of certainty (or confidence) in the body of evidence for each outcome assessed.	10–11, Table S7
**DISCUSSION**			
Discussion	23a	Provide a general interpretation of the results in the context of other evidence.	18–24
	23b	Discuss any limitations of the evidence included in the review.	24–25
	23c	Discuss any limitations of the review processes used.	24–25
	23d	Discuss implications of the results for practice, policy, and future research.	24–25
**OTHER INFORMATION**			
Registration and protocol	24a	Provide registration information for the review, including register name and registration number, or state that the review was not registered.	6
	24b	Indicate where the review protocol can be accessed, or state that a protocol was not prepared.	6
	24c	Describe and explain any amendments to information provided at registration or in the protocol.	9
Support	25	Describe sources of financial or non-financial support for the review, and the role of the funders or sponsors in the review.	Not applicable
Competing interests	26	Declare any competing interests of review authors.	26
Availability of data, code and other materials	27	Report which of the following are publicly available and where they can be found: template data collection forms; data extracted from included studies; data used for all analyses; analytic code; any other materials used in the review.	26

TABLE S2. Results of the search strategy used.

**Pubmed**		
nap* [tw] OR "daytime nap*" [tw] OR siesta [tw] OR "daytime sleep" [tw]	18,090	430
Heart Rate [Mesh] OR "heart rate" [tw] OR "heart rate variability" [tw] OR "Blood Pressure" [Mesh] OR "blood pressure" [tw] OR "Body Temperature" [Mesh] OR "body temperature" [tw] OR "biochemical reponse*" [tw] OR "hematological response*" OR "haematological response*" [tw] OR "muscle damage*" [tw] OR "Inflammation" [Mesh] OR inflammation* [tw] OR "Lactates" [Mesh] OR lactat* [tw] OR "blood lactate" [tw] OR "biomarkers of antioxidant defence*" [tw] OR "Oxidative Stress" [Mesh] OR "oxidative stress" [tw] OR "Electroencephalography" [Mesh] OR "Electroencephalography" [tw] OR Electroencephalogram* [tw] OR "Cardiac Output" [Mesh] OR "cardiac output*" [tw] OR "maximal oxygen uptake" [tw] OR $\dot{V}O_{2max}$ [tw] OR "Immunity" [Mesh] OR immun* [tw] OR "Physiology" [Mesh] OR physiolog* [tw] OR "physiological response*" [tw] OR "brain activit*" [tw] OR "perceived effort*" [tw] OR "perceived exertion*" [tw] OR "perceived fatigue" [tw] OR "Fatigue" [Mesh] OR fatigue* [tw] OR "rating of perceived exertion" [tw] OR RPE [tw] OR "perceived recovery*" [tw] OR "perceived recovery status" [tw] OR "muscle soreness*" [tw] OR "delayed onset muscle soreness" [tw] OR "Sleepiness" [Mesh] OR sleepiness [tw] OR "daytime sleepiness" [tw] OR "mood state*" [tw] OR mood [tw] OR feeling* [tw] OR "Emotions" [Mesh] OR emotion* [tw] OR "Depression" [Mesh] OR depression [tw] OR stress [tw] OR "Confusion" [Mesh] OR confusion [tw] OR "Anxiety" [Mesh] OR anxiety* [tw] OR tension [tw] OR "Anger" [Mesh] OR anger [tw] OR "Motivation" [Mesh] OR motivation* [tw] OR satisfaction* [tw] OR "Mental Health" [Mesh] OR "mental health" [tw] OR "Psychology" [Mesh] OR psychology [tw] OR "psychological response*" [tw] OR psychophysiological [tw] OR "psychophysiological response*" [tw]	9,139,066	
physical activit* [tw] OR "physically active*" [tw] OR athlete* [tw]	232,586	
**Web of science**		
napping OR nap OR "daytime nap" OR siesta OR "daytime sleep"	23,755	261
"heart rate" OR "heart rate variability" OR "blood pressure" OR "body temperature" OR "biochemical reponses" OR "hematological responses" OR "muscle damage" OR inflammation OR lactate OR "blood lactate" OR "biomarkers of antioxidant defence" OR "oxidative stress" OR "Electroencephalography" OR Electroencephalogram OR "cardiac output" OR "maximal oxygen uptake" OR $\dot{V}O_{2max}$ OR immunity OR physiology OR "physiological responses" OR "brain activity" OR "perceived effort" OR "perceived exertion" OR "perceived fatigue" OR fatigue OR "rating of perceived exertion" OR RPE OR "perceived recovery" OR "perceived recovery status" OR "muscle soreness" OR "delayed onset muscle soreness" OR sleepiness OR "daytime sleepiness" OR "mood state" OR mood OR feeling OR emotion OR depression OR stress OR confusion OR anxiety OR tension OR anger OR motivation OR satisfaction OR "mental health" OR psychology OR "psychological response" OR psychophysiological OR "psychophysiological response"	8,979,409	
"physical activity" OR "physically active" OR athletes	391,684	
**Scopus**		
napping OR nap OR "daytime nap*" OR siesta OR "daytime sleep"	27,889	769
"heart rate" OR "heart rate variability" OR "blood pressure" OR "body temperature" OR "biochemical reponses*" OR "hematological responses*" OR "muscle damage*" OR inflammation* OR lactat* OR "blood lactate*" OR "biomarkers of antioxidant defence*" OR "oxidative stress" OR "Electroencephalography" OR Electroencephalogram* OR "cardiac output*" OR "maximal oxygen uptake" OR $\dot{V}O_{2max}$ OR immunity* OR physiology* OR "physiological responses*" OR "brain activity*" OR "perceived effort*" OR "perceived exertion*" OR "perceived fatigue" OR fatigue* OR "rating of perceived exertion" OR RPE OR "perceived recovery*" OR "perceived recovery status" OR "muscle soreness*" OR "delayed onset muscle soreness" OR sleepiness OR "daytime sleepiness" OR "mood state*" OR mood OR feeling* OR emotion* OR depression OR stress OR confusion OR anxiety* OR tension OR anger OR motivation* OR satisfaction* OR "mental health" OR psychology OR "psychological response*" OR psychophysiological OR "psychophysiological response*"	12,270,543	
"physical activity*" OR "physically active*" OR athletes*	425,060	
**EMBASE**		
napping OR nap OR "daytime nap" OR siesta OR "daytime sleep"	11,993	262
"heart rate" OR "heart rate variability" OR "blood pressure" OR "body temperature" OR "biochemical reponses" OR "hematological responses" OR "muscle damage" OR inflammation OR lactate OR "blood lactate" OR "biomarkers of antioxidant defence" OR "oxidative stress" OR "Electroencephalography" OR Electroencephalogram OR "cardiac output" OR "maximal oxygen uptake" OR $\dot{V}O_{2max}$ OR immunity OR physiology OR "physiological responses" OR "brain activity" OR "perceived effort" OR "perceived exertion" OR "perceived fatigue" OR fatigue OR "rating of perceived exertion" OR RPE OR "perceived recovery" OR "perceived recovery status" OR "muscle soreness" OR "delayed onset muscle soreness" OR sleepiness OR "daytime sleepiness" OR "mood state" OR mood OR feeling OR emotion OR depression OR stress OR confusion OR anxiety OR tension OR anger OR motivation OR satisfaction OR "mental health" OR psychology OR "psychological response" OR psychophysiological OR "psychophysiological response"	8,561,133	
"physical activity" OR "physically active" OR athletes	391,463	
**ProQuest Central**		

TABLE S2. Continue.

napping OR nap OR “daytime nap” OR siesta OR “daytime sleep”	5,377	130
“heart rate” OR “heart rate variability” OR “blood pressure” OR “body temperature” OR “biochemical reponses” OR “hematological responses” OR “muscle damage” OR inflammation OR lactate OR “blood lactate” OR “biomarkers of antioxidant defence” OR “oxidative stress” OR “Electroencephalography” OR Electroencephalogram OR “cardiac output” OR “maximal oxygen uptake” OR $\dot{V}O_{2max}$ OR immunity OR physiology OR “physiological responses” OR “brain activity” OR “perceived effort” OR “perceived exertion” OR “perceived fatigue” OR fatigue OR “rating of perceived exertion” OR RPE OR “perceived recovery” OR “perceived recovery status” OR “muscle soreness” OR “delayed onset muscle soreness” OR sleepiness OR “daytime sleepiness” OR “mood state” OR mood OR feeling OR emotion OR depression OR stress OR confusion OR anxiety OR tension OR anger OR motivation OR satisfaction OR “mental health” OR psychology OR “psychological response” OR psychophysiological OR “psychophysiological response”	3,959,126	
“physical activity” OR “physically active” OR athletes	173,977	
**SPORTDiscus**		
napping OR nap OR “daytime nap” OR siesta OR “daytime sleep”	421	101
“heart rate” OR “heart rate variability” OR “blood pressure” OR “body temperature” OR “biochemical reponses” OR “hematological responses” OR “muscle damage” OR inflammation OR lactate OR “blood lactate” OR “biomarkers of antioxidant defence” OR “oxidative stress” OR “Electroencephalography” OR Electroencephalogram OR “cardiac output” OR “maximal oxygen uptake” OR $\dot{V}O_{2max}$ OR immunity OR physiology OR “physiological responses” OR “brain activity” OR “perceived effort” OR “perceived exertion” OR “perceived fatigue” OR fatigue OR “rating of perceived exertion” OR RPE OR “perceived recovery” OR “perceived recovery status” OR “muscle soreness” OR “delayed onset muscle soreness” OR sleepiness OR “daytime sleepiness” OR “mood state” OR mood OR feeling OR emotion OR depression OR stress OR confusion OR anxiety OR tension OR anger OR motivation OR satisfaction OR “mental health” OR psychology OR “psychological response” OR psychophysiological OR “psychophysiological response”	333,889	
“physical activity” OR “physically active” OR athletes	146,448	
**Cochrane**		
napping OR nap OR “daytime nap” OR siesta OR “daytime sleep”	6	2
“heart rate” OR “heart rate variability” OR “blood pressure” OR “body temperature” OR “biochemical reponses” OR “hematological responses” OR “muscle damage” OR inflammation OR lactate OR “blood lactate” OR “biomarkers of antioxidant defence” OR “oxidative stress” OR “Electroencephalography” OR Electroencephalogram OR “cardiac output” OR “maximal oxygen uptake” OR $\dot{V}O_{2max}$ OR immunity OR physiology OR “physiological responses” OR “brain activity” OR “perceived effort” OR “perceived exertion” OR “perceived fatigue” OR fatigue OR “rating of perceived exertion” OR RPE OR “perceived recovery” OR “perceived recovery status” OR “muscle soreness” OR “delayed onset muscle soreness” OR sleepiness OR “daytime sleepiness” OR “mood state” OR mood OR feeling OR emotion OR depression OR stress OR confusion OR anxiety OR tension OR anger OR motivation OR satisfaction OR “mental health” OR psychology OR “psychological response” OR psychophysiological OR “psychophysiological response”	4,513	
“physical activity” OR “physically active” OR athletes	214	
**PsycInfo**		
napping OR nap OR “daytime nap” OR siesta OR “daytime sleep”	2478	60
“heart rate” OR “heart rate variability” OR “blood pressure” OR “body temperature” OR “biochemical reponses” OR “hematological responses” OR “muscle damage” OR inflammation OR lactate OR “blood lactate” OR “biomarkers of antioxidant defence” OR “oxidative stress” OR “Electroencephalography” OR Electroencephalogram OR “cardiac output” OR “maximal oxygen uptake” OR $\dot{V}O_{2max}$ OR immunity OR physiology OR “physiological responses” OR “brain activity” OR “perceived effort” OR “perceived exertion” OR “perceived fatigue” OR fatigue OR “rating of perceived exertion” OR RPE OR “perceived recovery” OR “perceived recovery status” OR “muscle soreness” OR “delayed onset muscle soreness” OR sleepiness OR “daytime sleepiness” OR “mood state” OR mood OR feeling OR emotion OR depression OR stress OR confusion OR anxiety OR tension OR anger OR motivation OR satisfaction OR “mental health” OR psychology OR “psychological response” OR psychophysiological OR “psychophysiological response”	1971623	
“physical activity” OR “physically active” OR athletes	78305	
**Scielo**		
napping OR nap OR “daytime nap” OR siesta OR “daytime sleep”	110	1
“heart rate” OR “heart rate variability” OR “blood pressure” OR “body temperature” OR “biochemical reponses” OR “hematological responses” OR “muscle damage” OR inflammation OR lactate OR “blood lactate” OR “biomarkers of antioxidant defence” OR “oxidative stress” OR “Electroencephalography” OR Electroencephalogram OR “cardiac output” OR “maximal oxygen uptake” OR $\dot{V}O_{2max}$ OR immunity OR physiology OR “physiological responses” OR “brain activity” OR “perceived effort” OR “perceived exertion” OR “perceived fatigue” OR fatigue OR “rating of perceived exertion” OR RPE OR “perceived recovery” OR “perceived recovery status” OR “muscle soreness” OR “delayed onset muscle soreness” OR sleepiness OR “daytime sleepiness” OR “mood state” OR mood OR feeling OR emotion OR depression OR stress OR confusion OR anxiety OR tension OR anger OR motivation OR satisfaction OR “mental health” OR psychology OR “psychological response” OR psychophysiological OR “psychophysiological response”	96 307	
“physical activity” OR “physically active” OR athletes	9 980	

TABLE S3. The list of excluded studies during the full-text screening stage.

Studies	Exclusion reasons
Rachiwong, S., & Benjapalakorn, B. (2022). A 10-Minute Napping Can Help in Recovery in Motor Performance. Journal of Exercise Physiology Online, 25(3).	No physiological or perceptual measures after napping
Romyn, G., Lastella, M., Miller, D. J., Versey, N. G., Roach, G. D., & Sargent, C. (2018). Daytime naps can be used to supplement night-time sleep in athletes. Chronobiology international, 35(6), 865–868.	No physiological or perceptual measures after napping
Petit, E., Bourdin, H., Tio, G., Yenil, O., Haffen, E., & Mougin, F. (2018). Effects of a 20-min nap post normal and jet lag conditions on P300 components in athletes. International journal of sports medicine, 39(07), 508–516.	No physiological or perceptual measures after napping
Gupta, L., Morgan, K., North, C., & Gilchrist, S. (2021). Napping in high-performance athletes: Sleepiness or sleepability?. European Journal of Sport Science, 21(3), 321–330.	No physiological or perceptual measures after napping
Takahara, M., Nakamura, R., Ono, Y., & Oki, F. (2014). Effect of a 20-minute nap after sleep restriction on physical performance. International Journal of Psychophysiology, 2(94), 228–229.	Not athletes or physically active participants
Mograss, M., Frimpong, E., Vilcourt, F., Chouchou, F., Zvionow, T., & Dang-Vu, T. T. (2024). The effects of acute exercise and a nap on heart rate variability and memory in young sedentary adults. Psychophysiology, 61(2), e14454.	Not athletes or physically active participants
Mograss, M., Abi-Jaoude, J., Frimpong, E., Chalati, D., Moretto, U., Tarelli, L., … & Dang-Vu, T. T. (2022). The effects of napping on night-time sleep in healthy young adults. Journal of sleep research, 31(5), e13578.	Not athletes or physically active participants
Romyn, G., Roach, G. D., Lastella, M., Miller, D. J., Versey, N. G., & Sargent, C. (2022). The impact of sleep inertia on physical, cognitive, and subjective performance following a 1-or 2-hour afternoon nap in semiprofessional athletes. International Journal of Sports Physiology and Performance, 17(7), 1140–1150.	Study design
Pereira, R., Hartescu, I., Jackson, R. C., & Morgan, K. (2023). Napping behaviour, daytime sleepiness, and arousal in high performance athletes and non-athlete controls. Journal of Sports Sciences, 41(16), 1530–1537.	Study design
Knechtle, B., Wirth, A., Knechtle, P., Rüst, C. A., Rosemann, T., & Lepers, R. (2012). No improvement in race performance by naps in male ultra-endurance cyclists in a 600-km ultra-cycling race. Chin J Physiol, 55(2), 125–133.	Study design
Pereira, R., Hartescu, I., Jackson, R. C., & Morgan, K. (2023). Napping behaviour, daytime sleepiness, and arousal in high performance athletes and non-athlete controls. Journal of Sports Sciences, 41(16), 1530–1537.	Study design
Pointon, M., & Marino, F. (2013). The effect of "power naps" on aerobic capacity in sleep-deprived subjects. Journal of Science and Medicine in Sport, 16, e31.	Not eligible publication
Willmer, F., Reuter, C., Pramsohler, S., & Netzer, N. (2022). Napping improves wakefulness in athletes but has no influence on endurance performance. Sleep Medicine, 100, S181.	Not eligible publication
Souabni, M., Souabni, M. J., Hidouri, S., Ammar, A., Younes, M., Hammouda, O., & Driss, T. (2024). Napping and heart rate variability in elite athletes. Biology of Sport, 41(3), 213–221.	No available data
Ayhan, S. (2022). The effects of a 30-Minute Napping Opportunity after a night of partial sleep denied on cognitive and short-term high-intensity performance and Mood States. Pakistan Journal of Medical & Health Sciences, 16(02), 410–410.	No available data
Moore, J. P., Walsh, N. P., & Zurawlew, M. J. (2015). Daytime napping results in an underestimation of thermal strain during exercise in the heat. Occupational and environmental medicine, 72(10), 753–753.	Not an original article

TABLE S4. A summary of the 34 studies assessing psychophysiological measures after napping following normal sleep or sleep restriction in physically active individuals and athletes.

Study	Country	Sample size (sex)	Age (y)	Level of practice	Activity	Nocturnal sleep
Waterhouse et al. [18]	United Kingdom	10 (male)	23.3 ± 3.4	Physically active	NM	Partially deprived (sleep 2300–0300 h only)
Petit et al. [43]	France	16 (male)	22 ± 2	Athletes	NM	Normal (≈8 hours)
Pelka et al. [44]	Germany	27(male)	25 ± 1	Athletes	Individual sports (e.g., track and feld, tennis) (n=12) and team sports (e.g., football, handball) (n=15)	No sleep restriction (self-report)
Blanchfield et al. [45]	United Kingdom	11 (male)	35 ± 12	Athletes	Individual sport (i.e., running)	Normal sleep (≈7 hours)
Hammouda et al. [46]	Tunisia	9 (male)	18.5 ± 0.9	Athletes	Individual sports (i.e., Judo)	Partially deprived (4.5 hours)

Study	Nap duration (min)	Sleep assessment	Time of day of napping	Time allowed to avoid sleep inertia after napping (min)	Measured parameters	Results
Waterhouse et al. [18]	30	None	1300 h	30	HR	↓
					Temperature	↓
					Sleepiness	↓
Petit et al. [43]	20	PSG	1300 h	210	Temperature	NC
					Lactate	NC
Pelka et al. [44]	25	None	NM	NM	HR	NC
					Stress	NC
					Recovery	NC
Blanchfield et al. [45]	40	Actigraphy	NM	NM	Sleepiness	↑
					Temperature	NC
					Fatigue (BRUMS)	NC
					Vigour (BRUMS)	NC
					Total mood (BRUMS)	NC
					Urine-specific gravity	NC
					RPE	NC
					HR	NC
Hammouda et al. [46]	20	None	1410 h	30	LDH	NC
					WBC	NC
					RBC	NC
					MCV	↓ vs. CON
					MCH	↑ vs. CON
					PL	↑ vs. CON
					MPV	↑ vs. CON
					MG^{++}	↑ vs. CON
					LY	↑ vs. CON
					MO	↑ vs. CON
					HT	NC
					HB	NC
					NA^{+}	NC
					K^{+}	NC
					ALAT	NC
	90		1300 h		LDH	NC
					WBC	NC
					RBC	NC
					MCV	↓ vs. N20
					MCH	↓ vs. N20
					PL	NC
					MPV	↑ vs. CON and N20
					MG^{++}	↓ vs. N20
					LY	↑ vs. CON
					MO	↑ vs. CON and N20
					HT	↑ vs. CON
					HB	↑ vs. CON and N20
					NA^{+}	↑ vs. CON and N20
					K^{+}	↑ vs. CON and N20
					ALAT	↑ vs. CON

TABLE S4. Continue.

Study	Country	Sample size (sex)	Age (y)	Level of practice	Activity	Nocturnal sleep
O'Donnell et al. [20]	New Zealand	14 (female)	23 ± 6	Athletes	Team sport (i.e., netball)	No sleep restriction
Tanabe et al. [21]	Japan	7 (male)	21 ± 4	Physically active	NM	Normal sleep (≈8 hours)
Abdessalem et al. [47]	Tunisia	18 (male)	21 ± 3	Physically active	Individual sport (i.e., running)	Normal sleep (≈7 hours)
Boukhris et al. [48]	Tunisia	17 (male)	21 ± 3	Physically active	Individual sport (i.e., running)	Normal sleep (≈7 hours)
Brotherton et al. [19]	United Kingdom	15 (male)	22.7 ± 2.5	Athletes	Individual sports	Two nights partially deprived
Daaloul et al. [51]	Tunisia	13 (male)	23 ± 2	Athletes	Individual sport (i.e., karate)	Normal sleep
						Sleep deprivation
Hsouna et al. [53]	Tunisia	20 (male)	21 ± 4	Physically active	Individual sport (i.e., running)	Normal sleep (≈7 hours)

Study	Nap duration (min)	Sleep assessment	Time of day of napping	Time allowed to avoid sleep inertia after napping (min)	Measured parameters	Results
O'Donnell et al. [20]	$<$ 20	None	1330 h	30	Perceived performance	NC
					Energy levels	NC
	$>$ 20				Perceived performance	NC
					Energy levels	NC
Tanabe et al. [21]	30	PSG	1330 h	60	Sleepiness	NC
	60		1300 h		Sleepiness	NC
	90		1230 h		Sleepiness	NC
Abdessalem et al. [47]	25	None	13:00 h	215	RPE recorded during exercise	NC
			14:00 h	155	RPE recorded during exercise	NC
			15:00 h	95	RPE recorded during exercise	NC
Boukhris et al. [48]	25	Subjective sleep quality scale	1400 h	155	RPE recorded immediately after exercise	NC
					RPE recorded during exercise	NC
	35			145	RPE recorded immediately after exercise	NC
					RPE recorded during exercise	NC
	45			135	RPE recorded immediately after exercise	↓ vs. CON and N25
					RPE recorded during exercise	↓ vs. CON and N25
Brotherton et al. [19]	60	None	1300 h	180	Sleepiness	↓ vs. CON
					Temperature	NC
					Tiredness	↓ vs. CON
					Fatigue	↓ vs. CON
					Depression	↓ vs. CON
					Confusion	↓ vs. CON
					Happiness	↑ vs. CON
					Calm	↑ vs. CON
					Tension	NC
					Anger	↓ vs. CON
					Vigour	↑ vs. CON
Daaloul et al. [51]	30	Actigraphy	1300 h	30	Sleepiness	↓ vs. CON
					Fatigue	NC
					Sleepiness	↓ vs. CON
					Fatigue	NC
Hsouna et al. [53]	25	None	1400 h	155	Feelings score	NC
					Muscle soreness	NC
					Fatigue	↓ vs. CON
					Stress	↓ vs. CON
	35			145	Feelings score	NC
					Muscle soreness	NC
					Fatigue	↓ vs. CON
					Stress	↓ vs. CON
	45			135	Feelings score	NC
					Muscle soreness	NC
					Fatigue	↓ vs. CON, N25, and N35
					Stress	↓ vs. CON, N25, and N35

TABLE S4. Continue.

Study	Country	Sample size (sex)	Age (y)	Level of practice	Activity	Nocturnal sleep
Suppiah et al. [22]	Singapore	19 (male)	15 ± 1	Athletes	Individual sport (i.e., shooting sport)	Normal sleep (7 hours 45 min)
Ajjimaporn et al. [23]	Thailand	11 (male)	20 ± 1	Athletes	Team sports (i.e., football)	Partial sleep deprivation
Boukhris et al. [10]	Tunisia	14 (male)	20 ± 3	Athletes	Team sport	Normal sleep (≈8–9 hours)

Study	Nap duration (min)	Sleep assessment	Time of day of napping	Time allowed to avoid sleep inertia after napping (min)	Measured parameters	Results
Suppiah et al. [22]	30	A wire- less dry electro-encephalogram	45	1430 h	Sleepiness	↑ vs. CON
					Mean RR	NC
					RMSSD	NC
					HF power	NC
					LF power	NC
					LF/HF	NC
Ajjimaporn et al. [23]	20	Electro-encephalogram	100	1400 h	Fz delta	NC
					Fz theta	NC
					Fz alpha	NC
					Fz beta	NC
					Cz delta	NC
					Cz theta	NC
					Cz alpha	NC
					Cz beta	NC
					Pz delta	NC
					Pz theta	NC
					Pz alpha	NC
					Pz beta	NC
					RPE recorded immediately after exercise	NC
Boukhris et al. [10]	40	Actigraphy	1400 h	140	Sleepiness	↓ vs. CON
					Tension	↓ vs. CON
					Depression	↓ vs. CON
					Anger	↓ vs. CON
					Vigour	↑ vs. CON
					Fatigue	↓ vs. CON
					Confusion	NC
					POMS total score	↓ vs. CON
					Muscle soreness	↓ vs. CON
					Perceived recovery	↓ vs. CON
					RPE recorded during exercise	↓ vs. CON
					RPE recorded immediately after exercise	↓ vs. CON
	90			90	Sleepiness	↓ vs. CON and N40
					Tension	↓ vs. CON and N40
					Depression	↓ vs. CON and N40
					Anger	↓ vs. CON and N40
					Vigour	↑ vs. CON
					Fatigue	↓ vs. CON and N40
					Confusion	NC
					POMS total score	↓ vs. CON and N40
					Muscle soreness	↓ vs. CON and N40
					Perceived recovery	↓ vs. CON and N40
					RPE recorded during exercise	↓ vs. CON and N40
					RPE recorded immediately after exercise	↓ vs. CON and N40

TABLE S4. Continue.

Study	Country	Sample size (sex)	Age (y)	Level of practice	Activity	Nocturnal sleep
Romdhani et al. [58]	Tunisia	9 (male)	18.51 ± 0.93	Athletes	Individual sports	Sleep deprivation

Study	Nap duration (min)	Sleep assessment	Time of day of napping	Time allowed to avoid sleep inertia after napping (min)	Measured parameters	Results
Romdhani et al. [58]	20	None	1410 h	30	Sleepiness	↓ vs. CON
					Temperature	NC
					Hooper index	↓ vs. CON
					Anger	NC
					Confusion	↑ vs. CON
					Depression	NC
					Fatigue	NC
					Tension	NC
					Vigour	NC
					ASAT	↓ vs. CON after exercise
					URE	NC
					GPx	NC
					UA	↑ vs. CON after exercise
					SOD	NC
					CK	NC
					GLC	NC
					Lactate	↑ vs. CON
					RPE recorded during exercise	NC
	90		1300 h		Sleepiness	↓ vs. CON
					Temperature	↓ vs. CON and N20
					Hooper index	↓ vs. CON and N20
					Anger	↓ vs. N20
					Confusion	NC
					Depression	↓ vs. CON
					Fatigue	↓ vs. CON
					Tension	NC
					Vigour	NC
					ASAT	↓ vs. CON after exercise
					URE	↓ vs. CON and N20 after exercise
					GPx	↑ vs. CON and N20 after exercise
					UA	↑ vs. CON after exercise
					SOD	NC
					CK	NC
					GLC	NC
					Lactate	NC
					RPE recorded during exercise	↓ vs. N20

TABLE S4. Continue.

Study	Country	Sample size (sex)	Age (y)	Level of practice	Activity	Nocturnal sleep
Souissi et al. [59]	Tunisia	14 (male)	21 ± 2	Physically active	Individual sports	Normal sleep
						Sleep deprivation
Ammar et al. [60]	Germany	10 (male)	27 ± 3.5	Physically active	Individual sport	Normal sleep
Boukhris et al. [17]	Tunisia	15 (male)	20 ± 3	Athletes	Team sports	Normal sleep
Nishida et al. [61]	Japan	11 (male)	21 ± 1	Athletes	Team sport	Normal sleep
Romdhani et al. [54]	Tunisia	14 (male)	20 ± 1	Athletes	Individual sport	Normal sleep

Study	Nap duration (min)	Sleep assessment	Time of day of napping	Time allowed to avoid sleep inertia after napping (min)	Measured parameters	Results
Souissi et al. [59]	30	None	1300 h	270	Lactate before	NC
					Lactate after exercise	NC
					Lactate 3-min after exercise	NC
					Tension	↓ vs. CON
					Fatigue	↓ vs. CON
					Confusion	↓ vs. CON
					Depression	↓ vs. CON
					Vigour	NC
					Lactate before	NC
					Lactate after exercise	↓ vs. CON
					Lactate 3-min after exercise	NC
					Tension	↓ vs. CON
					Fatigue	↓ vs. CON
					Confusion	↓ vs. CON
					Depression	↓ vs. CON
					Vigour	↑ vs. CON
Ammar et al. [60]	60	Subjective sleep quality scale	1300 h	60	Sleepiness	↓ vs. CON
Boukhris et al. [17]	40	Actigraphy	1400 h	140	Sleepiness	↓ vs. CON
					CK	↓ vs. CON
					LDH	↓ vs. CON
					ASAT	↓ vs. CON
					ALAT	↓ vs. CON
					CRP	↓ vs. CON
					Muscle soreness	↓ vs. CON
					Perceived recovery	↓ vs. CON
					RPE recorded during exercise	↓ vs. CON
					RPE recorded immediately after exercise	↓ vs. CON
Nishida et al. [61]	20	Actiheart 5	1300 h	190	Sleepiness	NC
	60			150	sleepiness	NC
Romdhani et al. [54]	20	None	1410 h	30	Sleepiness	↓ vs. CON
					Temperature	↓ vs. CON
					POMS	↓ vs. CON
					Lactate	↑ vs. CON
					LDH	NC
					GLC	↑ vs. CON
					GPx	↑ vs. CON
					SOD	↑ vs. CON
					URE	NC
	90		1300 h		Sleepiness	↑ vs. N20
					Temperature	↓ vs. CON
					POMS	↑ vs. N20
					Lactate	↓ vs. N20
					LDH	↓ vs. CON and N20
					GLC	↑ vs. CON
					GPx	NC
					SOD	↑ vs. CON
					URE	NC

TABLE S4. Continue.

Study	Country	Sample size (sex)	Age (y)	Level of practice	Activity	Nocturnal sleep
Romdhani et al. [57]	Tunisia	9 (male)	18.78 ± 1.09	Athletes	Individual sport	Sleep deprivation
Romdhani et al. [55]	Tunisia	14 (male)	19.78 ± 1.41	Athletes	Individual sport	Normal sleep
		9 (male)				Sleep deprivation
Romdhani et al. [56]	Tunisia	14 (male)	20.43 ± 1.22	Athletes	Individual sport	Normal sleep
Boukhris et al. [11]	Tunisia	16 (male)	20 ± 3	Athletes	Team sport	Normal sleep

Study	Nap duration (min)	Sleep assessment	Time of day of napping	Time allowed to avoid sleep inertia after napping (min)	Measured parameters	Results
Romdhani et al. [57]	20	Subjective sleep quality scale	1410 h	30	Lactate	↑ vs. CON
					ASAT	↓ vs. CON after exercise
					CK	NC
					LDH	NC
					GLC	NC
					SOD	↑ vs. CON after exercise
					GPx	↑ vs. CON after exercise
					UA	↑ vs. CON after exercise
Romdhani et al. [55]	20	Subjective sleep quality	1410 h	30	Sleepiness	↓ vs. CON
					POMS	↓ vs. CON
					Hooper index	NC
					Sleepiness	↓ vs. CON
					POMS	NC
					Hooper index	↓ vs. CON
Romdhani et al. [56]	20	Subjective sleep quality scale	1410 h	30	Lactate	↑ vs. CON
					GLC	NC
					ASAT	NC
					CK	NC
					URE	NC
					GPx	NC
					SOD	↑ vs. CON
Boukhris et al. [11]	40	Actigraphy	1400 h	140	Sleepiness	↓ vs. CON
					Temperature	↓ vs. CON
					HR	↓ vs. CON during nap and exercise
					HRV score	↑ vs. CON during nap and exercise
					RR	↑ vs. CON during nap and exercise
					RMSSD	↓ vs. CON during nap and exercise
					SDNN	↑ vs. CON during nap and exercise
					PNN50	↑ vs. CON during nap and exercise
					HF	↑ vs. CON during nap and exercise
					LF	↑ vs. CON during nap and exercise
					HF/LF	↓ vs. CON during nap
					DBP	↓ vs. CON at all times
					SBP	↓ vs. CON at all times
					Perceived recovery	↓ vs. CON
					RPE recorded during exercise	↓ vs. CON
					RPE recorded immediately after exercise	↓ vs. CON

TABLE S4. Continue.

Study	Country	Sample size (sex)	Age (y)	Level of practice	Activity	Nocturnal sleep
Boukhris et al. [11]	Tunisia	16 (male)	20 ± 3	Athletes	Team sport	Normal sleep
Hsouna et al. [52]	Tunisia	12 (male)	23 ± 3	Athletes	Team sport	Normal sleep
Souabni et al. [63]	France	12 (male)	26 ± 5	Athletes	Team sport	Normal sleep
Bentouati et al. [65]	Tunisia	14 (male)	19.85 ± 2.07	Athletes	Individual sport	Norma sleep

Study	Nap duration (min)	Sleep assessment	Time of day of napping	Time allowed to avoid sleep inertia after napping (min)	Measured parameters	Results
Boukhris et al. [11]	90	Actigraphy	1400 h	90	Sleepiness	↓ vs. CON and N40
					Temperature	↓ vs. CON and N40
					HR	↓ vs. CON and N40 during nap and exercise
					HRV score	↑ vs. CON and N40 during nap and exercise
					RR	↑ vs. CON and N40 during nap and exercise
					RMSSD	↑ vs. CON and N40 during nap and exercise
					SDNN	↑ vs. CON and N40 during nap and exercise
					PNN50	↑ vs. CON and N40 during nap and exercise
					HF	↑ vs. CON and N40 during nap and exercise
					LF	↑ vs. CON during nap and exercise
					HF/LF	↓ vs. CON during nap and exercise
					DBP	↓ vs. CON and N40 at all times
					SBP	↓ vs. CON and N40 at all times
					Perceived recovery	↓ vs. CON and N40
					RPE recorded during exercise	↓ vs. CON and N40
					RPE recorded immediately after exercise	↓ vs. CON and N40
Hsouna et al. [52]	40	Actigraphy	1400 h	140	Sleepiness	↓ vs. CON
					Muscle soreness	↓ vs. CON
					RPE recorded during exercise	↓ vs. CON
Souabni et al. [63]	40	Actigraphy	1300 h	80	Sleepiness	↓ vs. CON
					Stress	↓ vs. CON
					Muscle soreness	NC
					Fatigue	NC
					Total Hooper score	NC
					HR_{mean}	↓ vs. CON during SSG
					HR_{peak}	↓ vs. CON during SSG and retest of SST
					RPE	↓ vs. CON during SSG and retest of SST
Bentouati et al. [65]	30	Subjective sleep quality scale	1300 h	30	RPE recorded immediately After exercise	↓ vs. CON
					Sleepiness	↓ vs. CON
					POMS	↓ vs. CON

TABLE S4. Continue.

Study	Country	Sample size (sex)	Age (y)	Level of practice	Activity	Nocturnal sleep
Boukhris et al. [50]	Tunisia	17 (male)	20 ± 3	Athletes	Team sports	Normal sleep
Souabni et al. [64]	France	10 (male)	27.6 ± 4.7	Athletes	Team sport	Normal sleep
Nishida et al. [62]	Japan	14 (male)	21.6 ± 0.5	Athletes	Team sport	Normal sleep
Teece et al. [66]	New Zealand	15 (male)	21 ± 2	Athletes	Team sport	Normal sleep
Willmer et al. [67]	Austria	12 (seven female and five male)	27.5 ± 2.8	Athletes	NM	Sleep deprivation
Kurtoğlu et al. [68]	Turkey	12 (male)	12.5 ± 1.31	Athletes	Individual sport	Normal sleep

Study	Nap duration (min)	Sleep assessment	Time of day of napping	Time allowed to avoid sleep inertia after napping (min)	Measured parameters	Results
Boukhris et al. [50]	40	Actigraphy and subjective sleep quality scale	1400 h	140	RPE recorded during exercise	↓ vs. CON
					RPE recorded immediately after exercise	↓ vs. CON
					Sleepiness	↓ vs. CON
					Perceived recovery	↑ vs. CON
					RMS	↑ vs. CON
					M_{max}	↑ vs. CON
					Ptw	↑ vs. CON
					VAL	↑ vs. CON
Souabni et al. [64]	40	Actigraphy and subjective sleep quality scale	1300 h	125	Temperature	↓ vs. CON
					Anger	NC
					Anxiety	↓ vs. CON
					Confusion	NC
					Depression	NC
					Fatigue	↓ vs. CON
					Vigour	↑ vs. CON
					HR_{mean}	↓ vs. CON
					HR_{peak}	↓ vs. CON
					RPE immediately after exercise	↓ vs. CON
Nishida et al. [62]	40	A smartphone application (Sleep Meister)	1400 h	0	Sleepiness	NC
					Fatigue	NC
Teece et al. [66]	60	Actigraphy	1200 h	30	Fatigue	↓ vs. CON
					Muscle soreness	NC
					Alertness	NC
Willmer et al. [67]	30	Polysomnography	1100 h	0	Sleepiness	↓ vs. CON
Kurtoğlu et al. [68]	25	None	1400 h	0	FVC	NC
					FEV1	NC
					FEV1/FVC	NC
					PEF	NC
					FEF25/75	NC
					FET	NC
	45				FVC	NC
					FEV1	NC
					FEV1/FVC	NC
					PEF	↑ vs. CON and N25
					FEF25/75	NC
					FET	NC

TABLE S4. Continue.

Study	Country	Sample size (sex)	Age (y)	Level of practice	Activity	Nocturnal sleep
Gallagher et al. [69]	United Kingdom	15 (male)	22 ± 1.59	Physically active	Individual sport	Sleep deprivation

↑: increase; ↓: decrease; HR: heart rate; PSG: polysomnography; NC: no change; NM: not mentioned; BRUMS: Brunel Mood Scale Questionnaire; RPE: rating of perceived exertion; LDH: lactate dehydrogenase; WBC: white blood cells; RBC: red blood cells; MCV: mean corpuscular volume; MCH: mean corpuscular hemoglobin; PL: platelets; MPV: mean platelet volume; Mg^{++}: magnesium ion; LY: lymphocytes; MO: monocytes; HT: hematocrit; HB: hemoglobin; Na^{+}: sodium ion; K^{+}: potassium ion; ALAT: alanine aminotransferase; POMS: profile of mood states; CON: control condition; N25: 25-min nap; N40: 40-min nap; HRV: heart rate variability; RR: the mean RR interval; RMSSD: the root mean square of successive R-R intervals; SDNN: the standard deviation of normal to normal intervals; PNN50: the percentage of RR intervals with a difference of duration greater than 50 ms; HF: High frequency; LF: Low frequency; DPB: diastolic blood pressure; SBP: systolic blood pressure; Fz: the midline frontal, Cz: the midline central and Pz; the midline parietal; ASAT: aspartate aminotransferase; URE: urea; GPX: glutathione peroxidase; UA: uric acid; SOD: superoxide dismutase; CK: creatine kinase; GLC: glycemia; CRP: C-reactive protein; FVC: forced vital capacity; PEF: maximum expiratory flow rate; FEV1: forced expiratory volume İn 1 s; PEF: peak expiratory flow; FET: forced expiratory time; RMS: root mean square; M_{max}: peak-to-peak M-wave amplitudes; Ptw: potentiated twitch; VAL: voluntary activation level.

Study	Nap duration (min)	Sleep assessment	Time of day of napping	Time allowed to avoid sleep inertia after napping (min)	Measured parameters	Results
Gallagher et al. [69]	30	None	13 h 00	30	Sleepiness	NC
					Temperature	↑ vs. CON
					Tiredness	NC
					Fatigue	NC
					Depression	NC
					Confusion	NC
					Happiness	NC
					Calm	NC
					Tension	NC
					Anger	NC
					Vigour	NC
	60			0	Sleepiness	NC
					Temperature	↑ vs. CON
					Tiredness	NC
					Fatigue	NC
					Depression	NC
					Confusion	NC
					Happiness	NC
					Calm	NC
					Tension	NC
					Anger	NC
					Vigour	NC

TABLE S5. The heterogeneity statistics for each outcome.

	Outcome	Q	p-value	$\tau^2_{Level\ 2}$	$\tau^2_{Level\ 3}$	$I^2_{Level\ 2}$ (within-clusters heterogeneity)	$I^2_{Level\ 3}$ (between-cluster heterogeneity	Overall I^2
Normal sleep	Sleepiness	304.28	< 0.001	0.14	6.11	2.14%	94.46%	96.6%
	Fatigue	76.52	< 0.001	0.29	0.57	28.32%	55.75%	83.42%
	Total mood score of POMS	6.55	0.365	0.01	0	3.05%	0%	3.05%
	RPE during exercise	99.79	< 0.01	0.16	1.74	7.68%	84.47%	92.15%
	RPE after exercise	54.41	< 0.001	0.13	0.50	16.95%	63.12%	80.07%
	Perceived recovery	54.41	< 0.001	0.13	0.50	16.95%	63.12%	80.07%
	Muscle soreness	39.50	< 0.001	0.68	0.28	60.35%	25.03%	85.37%
	HR during exercise	20.63	0.002	0	0.41	6.89%	7.33%	73.31%
	Temperature	44.29	< 0.001	0	1.49	0%	89.49%	89.49%
Sleep deprivation	Sleepiness	64.51	< 0.001	0	2.26	0%	84.91%	84.91%
	Fatigue	64.51	< 0.001	0	1.1	0%	91.5%	91.05%

HR: heart rate, POMS: Profile of mood states.

TABLE S6. Risk of bias assessment of the included studies.

Study	Randomisation process	Deviations from intended interventions	Missing outcome data	Measurement of the outcome	Selection of the reported result	Overall bias
**Waterhouse et al. [18]**	Some concerns	Some concerns	Low risk	Some concerns	Some concerns	Some concerns
**Petit et al. [43]**	Low risk	Low risk	Low risk	Low risk	Some concerns	Some concerns
**Pelka et al. [44]**	Low risk	Low risk	Low risk	Low risk	Some concerns	Some concerns
**Blanchfield et al. [45]**	Low risk	Low risk	Low risk	Some concerns	Some concerns	Some concerns
**Hammouda et al. [46]**	Some concerns	Some concerns	Low risk	Some concerns	Some concerns	Some concerns
**Daaloul et al. [51]**	Some concerns	Low risk	Low risk	Some concerns	Some concerns	Some concerns
**O'Donnell et al. [20]**	High risk	Some concerns	Some concerns	High risk	Some concerns	High risk
**Suppiah et al. [22]**	Low risk	Low risk	Low risk	Low risk	Some concerns	Some concerns
**Tanade et al. [21]**	Some concerns	Low risk	Low risk	Some concerns	Some concerns	Some concerns
**Abdessalem et al. [47]**	Low risk	Low risk	Low risk	Some concerns	Some concerns	Some concerns
**Boukhris et al. [48]**	Low risk	Low risk	Low risk	Some concerns	Some concerns	Some concerns
**Brotherton et al. [19]**	Low risk	Low risk	Low risk	Some concerns	Some concerns	Some concerns
**Hsouna et al. [53]**	Low risk	Some concerns	Low risk	Low risk	Some concerns	Some concerns
**Ajjimaporn et al. [23]**	Some concerns	Some concerns	Low risk	Some concerns	Some concerns	Some concerns
**Boukhris et al. [10]**	Some concerns	Some concerns	Low risk	Some concerns	Some concerns	Some concerns
**Romdhani et al. [58]**	Some concerns	Low risk	Low risk	High risk	Some concerns	High risk
**Souissi et al. [59]**	High risk	Some concerns	Low risk	High risk	Some concerns	High risk
**Ammar et al. [60]**	Some concerns	Some concerns	Low risk	Low risk	Some concerns	Some concerns
**Boukhris et al. [17]**	Low risk	Low risk	Low risk	Low risk	Some concerns	Some concerns
**Nishida et al. [61]**	Low risk	Low risk	Low risk	High risk	Some concerns	High risk
**Romdhani et al. [54]**	Low risk	Low risk	Low risk	Low risk	Some concerns	Some concerns
**Romdhani et al. [57]**	Low risk	Low risk	Low risk	Low risk	Some concerns	Some concerns
**Romdhani et al. [55]**	Low risk	Low risk	Low risk	Low risk	Some concerns	Some concerns
**Romdhani et al. [56]**	Low risk	Low risk	Low risk	Low risk	Some concerns	Some concerns
**Boukhris et al. [11]**	Some concerns	Low risk	Low risk	Some concerns	Some concerns	Some concerns
**Hsouna et al. [52]**	High risk	Some concerns	Low risk	Some concerns	Some concerns	High risk
**Souabni et al. [63]**	Some concerns	Low risk	Low risk	Low risk	Some concerns	Some concerns
**Bentouati et al. [65]**	Some concerns	Low risk	Low risk	Some concerns	Some concerns	Some concerns
**Boukhris et al. [50]**	Low risk	Some concerns	Low risk	Low risk	Some concerns	Some concerns
**Souabni et al. [64]**	Some concerns	Low risk	Low risk	Low risk	Some concerns	Some concerns
**Nishida et al. [62]**	Low risk	Low risk	Low risk	Some concerns	Some concerns	Some concerns
**Teece et al. [66]**	Low risk	Low risk	Low risk	Low risk	Some concerns	Some concerns
**Willmer et al. [67]**	Low risk	Some concerns	Low risk	Low risk	Some concerns	Some concerns
**Kurtoğlu et al. [68]**	Some concerns	Some concerns	Some concerns	Low risk	Some risk	Some concerns
**Gallagher et al. [69]**	Low risk	Low risk	Low risk	Some concerns	Some concerns	Some concerns

TABLE S7. Summary of findings according to the GRADE framework for the quality of evidence.

	Outcome	Certainty assessment							Number of participants	Effect	Certainty
		Number of studies (N ESs)	Study design	Risk of bias	Inconsistency	Indirectness	Imprecision	Publication bias		Absolute 95% CI	
Normal sleep	Sleepiness	17 (23)	Crossover randomised design[a]	Serious[b]	Very Serious[c]	Not serious	Very serious[d]	No	225	SMD = 1.09[e] 95% CI = -0.25–2.44	Low
	Fatigue	8 (11)	Crossover randomised design[a]	Serious[b]	Not serious[f]	Not serious	Very serious[d]	No	123	SMD = 0.91[e] 95% CI = 0.06–1.66	Low
	Mood states	5 (7)	Crossover randomised design[a]	Serious[b]	Not serious[g]	Not serious	Very serious[d]	No	67	SMD = 0.61 95% CI = 0.22–0.99	Very low
	RPE during exercise*	8 (16)	Crossover randomised design[a]	Serious[b]	Not serious[h]	Not serious	Very serious[d]	Yes	121	SMD = 1.62[e] 95% CI = 0.49–2.75	Moderate
	RPE after exercise*	7 (11)	Crossover randomised design[a]	Serious[b]	Not serious[i]	Not serious	Very serious[d]	Yes	103	SMD = 1.11[e] 95% CI = 0.38–1.84	Moderate
	Perceived recovery	5 (7)	Crossover randomised design[a]	Serious[b]	Not serious[g]	Not serious	Very serious[d]	Yes	89	SMD = 1.66[e] 95% CI = 0.59–2.73	Low
	Muscle soreness	6 (9)	Crossover randomised design[a]	Serious[b]	Not serious[j]	Not serious	Very serious[d]	Yes	88	SMD = 1.57[e] 95% CI = -0.13–3.27	Low
	Body temperature	5 (7)	Crossover randomised design[a]	Serious[b]	Not serious[k]	Not serious	Very serious[d]	No	67	SMD = 0.66 95% CI = -0.56–1.87	Very low
	HR during exercise	4 (7)	Crossover randomised design[a]	Serious[b]	Not serious[l]	Not serious	Very serious[d]	No	65	SMD = 0.62 95% CI = -0.21–1.45	Very low
Sleep deprivation	Sleepiness	7 (9)	Crossover randomised design[a]	Serious[b]	Not serious[m]	Not serious	Very serious[d]	Yes	68	SMD = 1.03[e] 95% CI = -0.31–2.36	Low
	Fatigue	7 (10)	Crossover randomised design[a]	Serious[b]	Not serious[n]	Not serious	Very serious[d]	No	66	SMD = 0.79 95% CI = -0.17–1.66	Very low

CI: confidence interval; SMD: standardized mean difference; ES: effect size; HR: Heart rate. **Explanations** a. Low level of evidence because of the experimental design nature of the included studies.; b. All included studies had some bias concerns.; c. The results were heterogeneous, with an I^2 value exceeding 75%.; d. A downgrade was performed because the total number of participants < 400, and wide CI.; e. An upgrade was performed because the large ES.; f. 9 ESs showed significant reduction in fatigue after napping, and one showed no significant effect of napping on fatigue.; g. The results were heterogeneous, with an I^2 value exceeding 75%.; h. Despite significant heterogeneity (I^2 > 75%), meta-regression and subgroup analyses identified nap duration, level of practice, and activity type as significant moderators, explaining a substantial portion of the variability, and 11 out of 16 ESs consistently showed reduced RPE after napping.; i. Despite significant heterogeneity (I^2 > 75%), meta-regression and subgroup analyses identified nap duration and activity type as significant moderators, explaining a substantial portion of the variability, and 8 out of 11 ESs consistently showed reduced RPE after napping.; g. Only one study showed non-significant effect of napping, and the remaining studies showed that napping improved significantly perceived recovery.; j. 7 out of 9 ESs showed significant reduction in muscle soreness after napping.; k. 5 out of 7 ESs showed significant decrease in body temperature after napping.; l. 6 out of 7 ESs showed significant decrease in HR during exercise after napping.; m. 6 ou of 9 ESs showed reduction in sleepiness after napping.; n. 8 ou of 10 ESs showed reduction in fatigue after napping.; * An upgrade was performed to due dose-response relationship (nap duration).

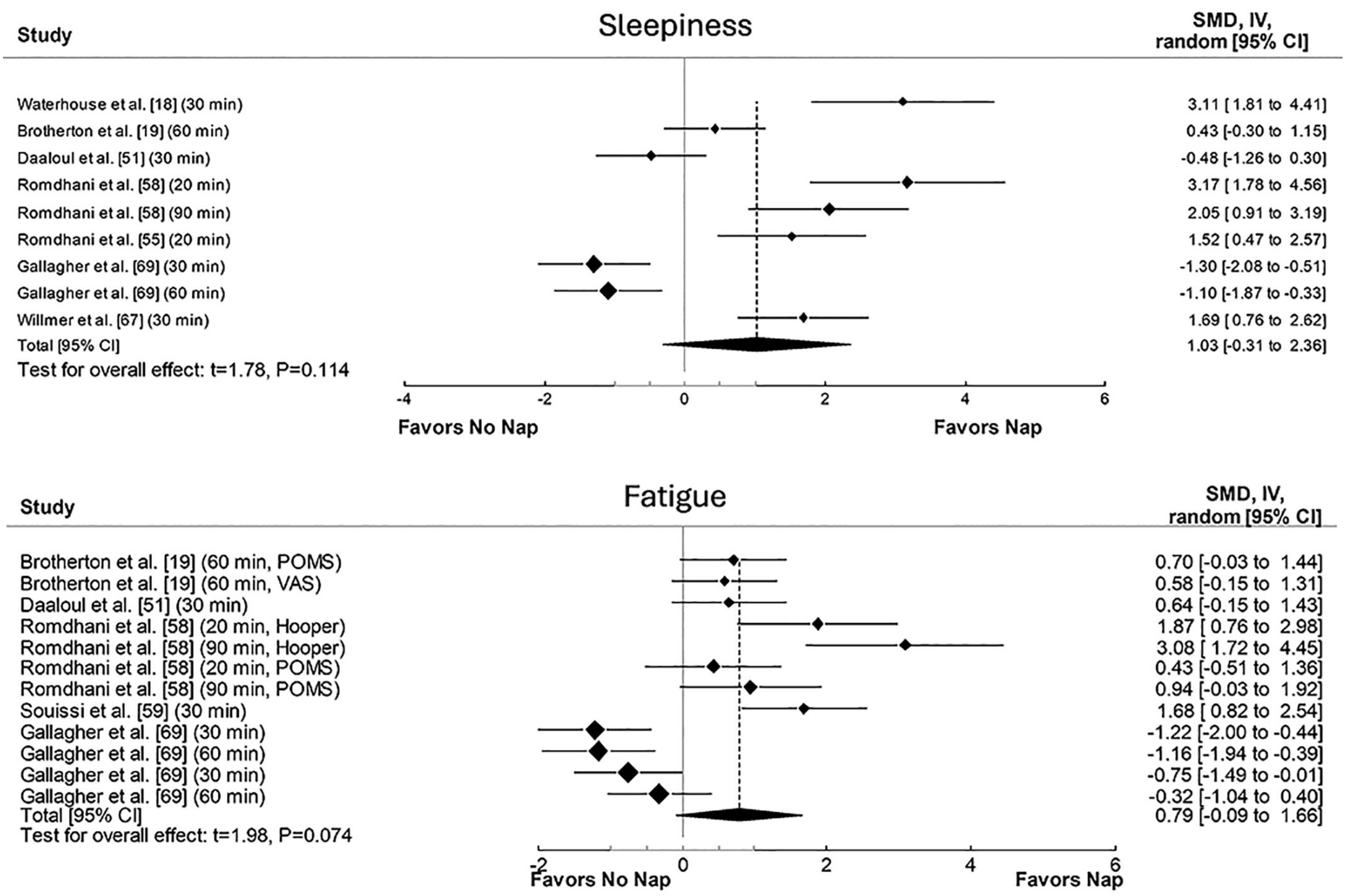

FIG. S1. Forest plot for the impacts of napping following sleep deprivation on sleepiness and fatigue. SMD: standardized mean difference, CI: confidence intervals.

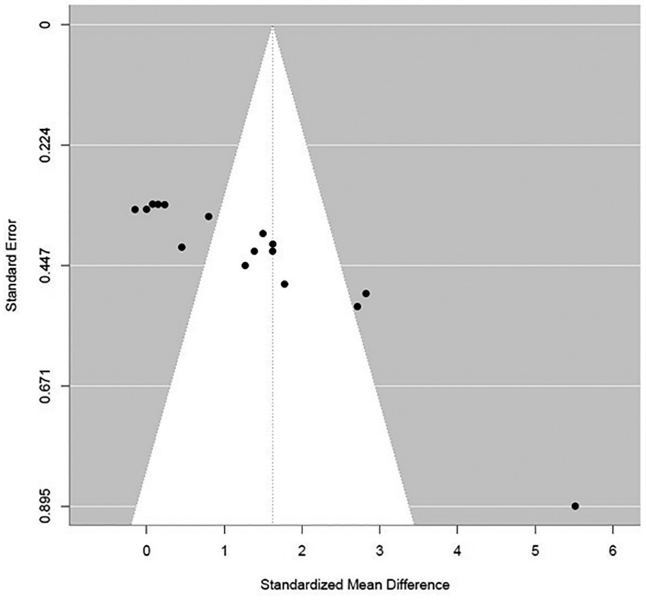

FIG. S2. Publications bias in RPE during exercise.

Publication bias for RPE during exercise following normal sleep

The visual inspection of the funnel plot (Figure S2) indicated evidence of publication bias, confirmed by the Begg and Mazumdar's rank correlation test bias (Kendall's tau = 0.68, $p < 0.0001$) and the multilevel model of Egger's test ($F_{(1, 14)} = 50.51$, $p < 0.0001$).

According to the Cook's distances, Hsouna, 2022 (40 min) was considered to be an outlier or overly influential for RPE post exercise.

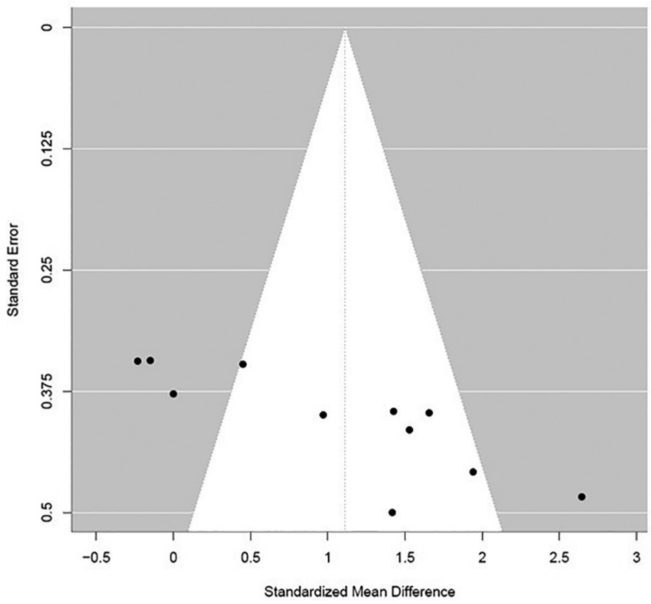

FIG. S3. Publication bias in RPE after exercise.

Publication bias for RPE after exercise following normal sleep

The visual inspection of the funnel plot (Figure S3) indicated evidence of publication bias, confirmed by the Begg and Mazumdar's rank correlation test bias (Kendall's tau = 0.64, p = 0.0057) and the multilevel model of Egger's test ($F_{(1,9)}$ = 12.92, p = 0.0058).

According to the Cook's distances, none of the studies were considered to be an outlier or overly influential for RPE post exercise.

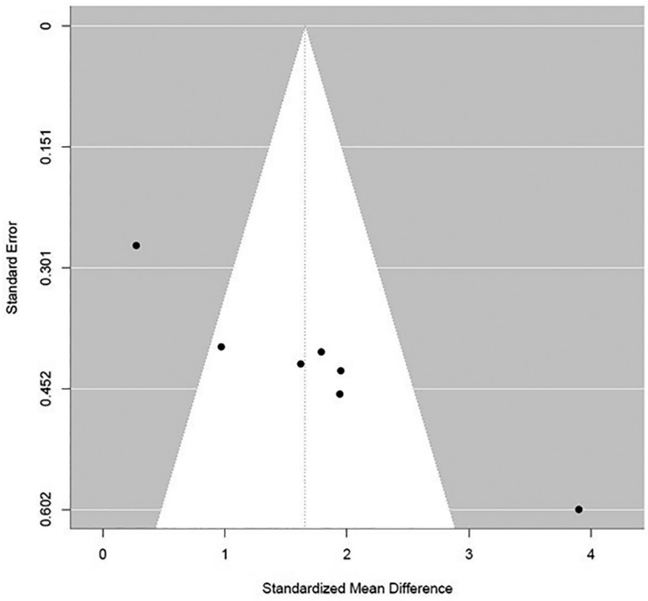

FIG. S4. Publication bias in perceived recovery.

Publication bias for perceived recovery following normal sleep

The visual inspection of the funnel plot (Figure S4) indicated evidence of publication bias, confirmed by the Begg and Mazumdar's rank correlation test bias (Kendall's tau = -0.81, p = 0.011) and the multilevel model of Egger's test ($F_{(1,5)}$ = 36.8, p = 0.002).

Cook's distances indicated that none of the studies were considered to be an outlier or overly influential for recovery.

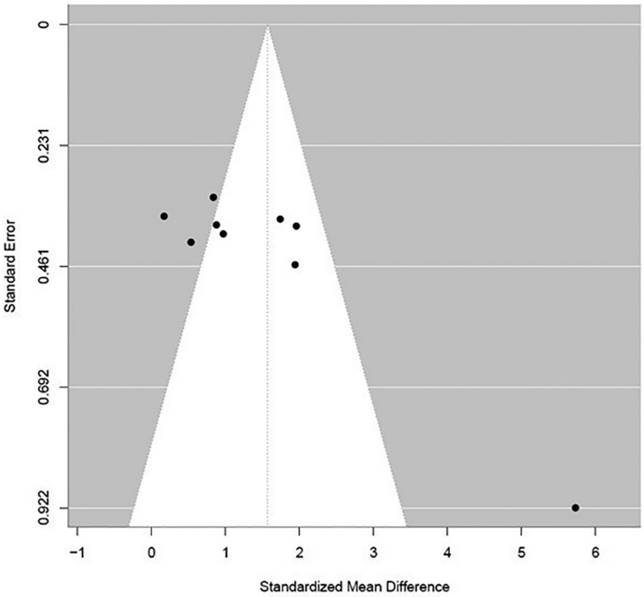

FIG. S5. Publication bias in muscle soreness.

Publication bias for muscle soreness following normal sleep

The visual inspection of the funnel plot (Figure S5) indicated evidence of publication bias, confirmed by the Begg and Mazumdar's rank correlation test bias (Kendall's tau = -0.81, p = 0.011) and the multilevel model of Egger's test ($F_{(1,5)} = 36.8$, $p = 0.002$). Cook's distances indicated that none of the studies were considered to be an outlier or overly influential for recovery.

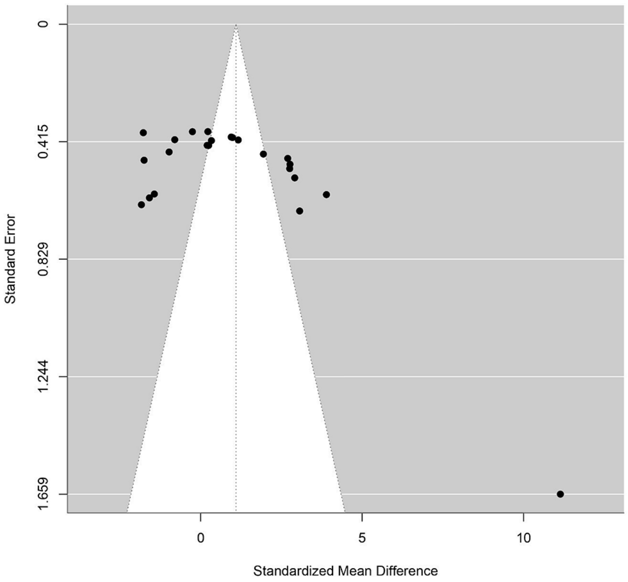

FIG. S6. Publication bias in sleepiness.

Publication bias for sleepiness following normal sleep

The visual inspection of the funnel plot (Figure S6) indicated evidence of publication bias, confirmed by the multilevel model of Egger's test ($F_{(1,21)} = 26.66$, $p < 0.0001$). However, the Begg and Mazumdar's rank correlation test showed no evidence of publication bias (Kendall's tau = 0.23, p = 0.127).

According to the Cook's distances, Hsouna, 2022 (40 min) was considered to be an outlier or overly influential for Sleepiness.

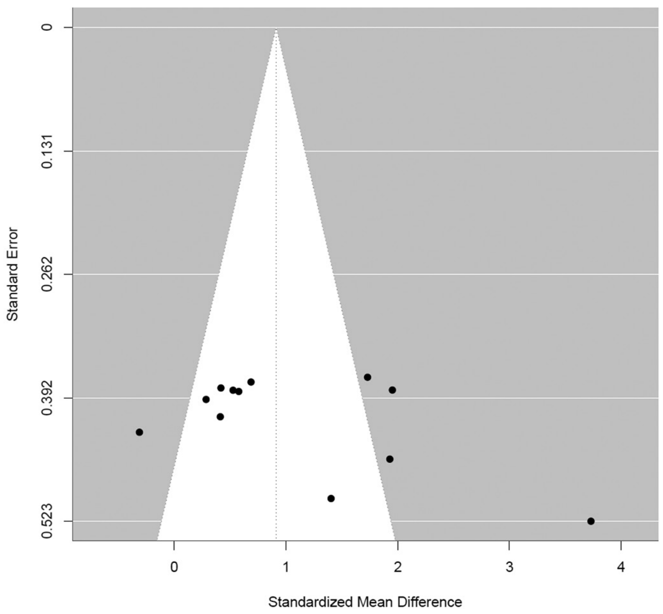

FIG. S7. Publication bias in fatigue.

Publication bias for fatigue following normal sleep

The visual inspection of the funnel plot (Figure S7) indicated evidence of publication bias, confirmed by the multilevel model of Egger's test ($F_{(1, 10)} = 11.38$, $p = 0.0071$). However, the Begg and Mazumdar's rank correlation test showed no evidence of publication bias (Kendall's tau = 0.03, $p = 0.946$).
Cook's distances indicated that none of the studies were considered to be an outlier or overly influential for fatigue.

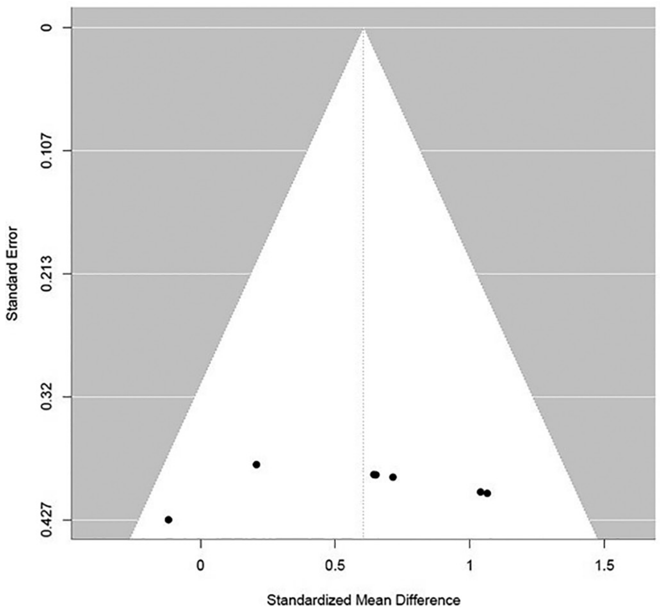

FIG. S8. Publication bias in mood states.

Publication bias for total mood score of POMS following normal sleep

The visual inspection of the funnel plot (Figure S8) indicated no evidence of publication bias, confirmed by the multilevel model of Egger's test ($F_{(1, 5)} = 0.2$, $p = 0.67$) and the Begg and Mazumdar's rank correlation test showed no evidence of publication bias (Kendall's tau = 0.43, $p = 0.239$).
Cook's distances indicated that none of the studies were considered to be an outlier or overly influential for POMS.

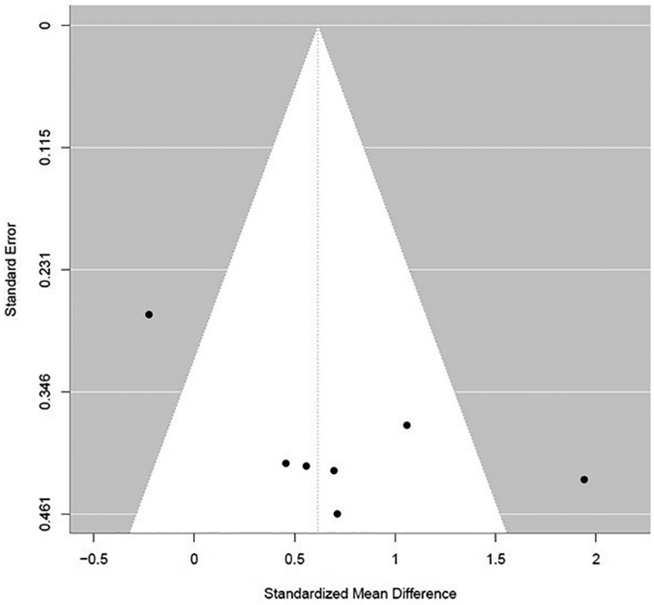

FIG. S9. Publication bias in HR during exercise.

Publication bias for HR during the exercise following normal sleep

The visual inspection of the funnel plot (Figure S9) indicated no evidence of publication bias, confirmed by the multilevel model of Egger's test ($F_{(1, 5)} = 3.5$, $p = 0.12$) and the Begg and Mazumdar's rank correlation test showed no evidence of publication bias (Kendall's tau = 0.52, p = 0.136).

Cook's distances indicated that Pelka (2017) (25 min) was considered to be an outlier or overly influential for HR during the exercise.

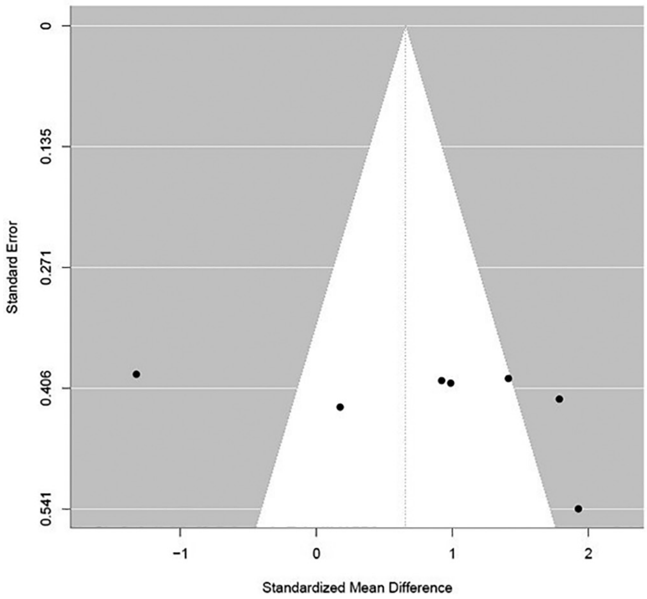

FIG. S10. Publication bias in body temperature.

Publication bias for body temperature following normal sleep

The visual inspection of the funnel plot (Figure S10) indicated no evidence of publication bias, confirmed by the multilevel model of Egger's test ($F_{(1, 5)} = 1.88$, $p = 0.23$) and the Begg and Mazumdar's rank correlation test showed no evidence of publication bias (Kendall's tau = 0.33, p = 0.381).

Cook's distances indicated that Petit et al. (2014) (20 min) was considered to be an outlier or overly influential for temperature.

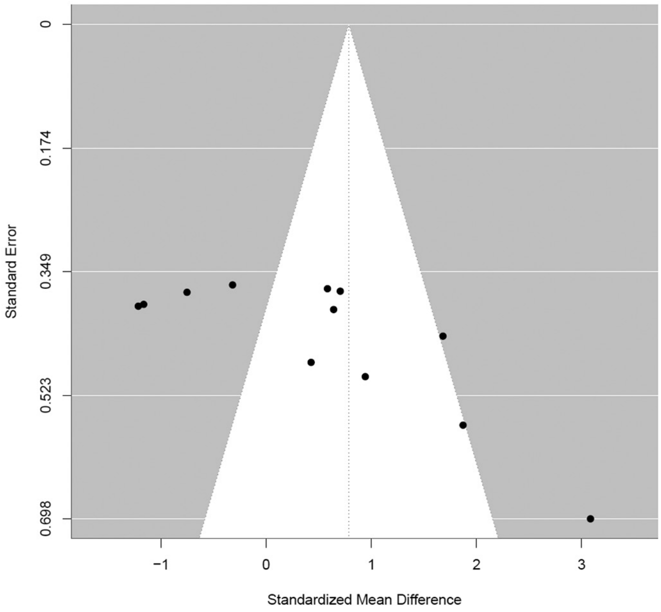

FIG. S11. Publication bias in fatigue.

Publication bias for fatigue following sleep deprivation

The visual inspection of the funnel plot (Figure S11) indicated evidence of publication bias confirmed by the multilevel model of Egger's test ($F_{(1,10)} = 5.45$, $p = 0.04$). However, the Begg and Mazumdar's rank correlation test bias (Kendall's tau = 0.42, p = 0.062) and showed no evidence of publication.

Cook's distances indicated no studies in this meta-analysis may be overly influential on the fatigue results.

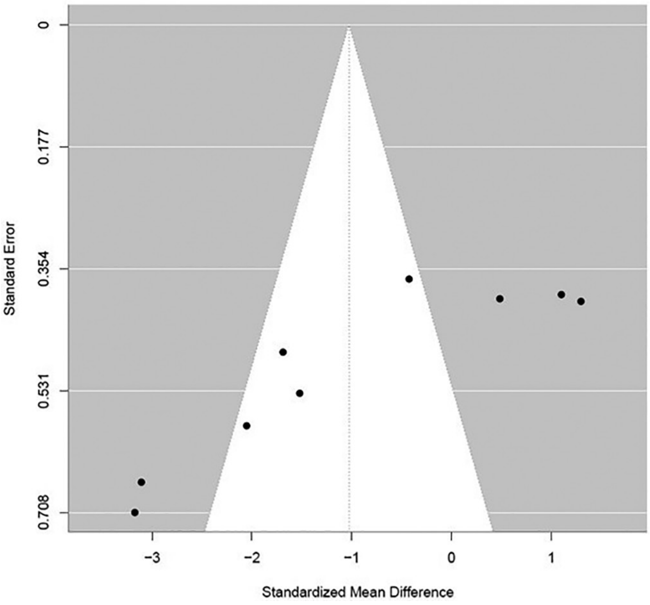

FIG. S12. Publication bias in sleepiness.

Publication bias for sleepiness following sleep deprivation

The visual inspection of the funnel plot (Figure S12) indicated evidence of publication bias, which confirmed by the Begg and Mazumdar's rank correlation test bias (Kendall's tau = 0.61, p = 0.025) and the multilevel model of Egger's test ($F_{(1,7)} = 17.85$, $p = 0.004$) showed no evidence of publication.

Cook's distances indicated no studies in this meta-analysis may be overly influential on the sleepiness results.
